# Imputation to whole-genome sequence and its use in genome-wide association studies for pork colour traits in crossbred and purebred pigs

**DOI:** 10.3389/fgene.2022.1022681

**Published:** 2022-10-11

**Authors:** Marzieh Heidaritabar, Abe Huisman, Kirill Krivushin, Paul Stothard, Elda Dervishi, Patrick Charagu, Marco C. A. M. Bink, Graham S. Plastow

**Affiliations:** ^1^ Department of Agricultural, Food and Nutritional Science, University of Alberta, Edmonton, AB, Canada; ^2^ Hendrix Genetics Research, Boxmeer, Netherlands; ^3^ Hendrix Genetics, Business Unit Swine, Regina, SK, Canada

**Keywords:** pork color traits, crossbred pigs, purebred pigs, imputed whole-genome sequence, GWAS, QTL regions

## Abstract

Imputed whole-genome sequence (WGS) has been proposed to improve genome-wide association studies (GWAS), since all causative mutations responsible for phenotypic variation are expected to be present in the data. This approach was applied on a large number of purebred (PB) and crossbred (CB) pigs for 18 pork color traits to evaluate the impact of using imputed WGS relative to medium-density marker panels. The traits included Minolta A*, B*, and L* for fat (FCOL), quadriceps femoris muscle (QFCOL), thawed loin muscle (TMCOL), fresh ham gluteus medius (GMCOL), ham iliopsoas muscle (ICOL), and longissimus dorsi muscle on the fresh loin (FMCOL). Sequence variants were imputed from a medium-density marker panel (61K for CBs and 50K for PBs) in all genotyped pigs using BeagleV5.0. We obtained high imputation accuracy (average of 0.97 for PBs and 0.91 for CBs). GWAS were conducted for three datasets: 954 CBs and 891 PBs, and the combined CBs and PBs. For most traits, no significant associations were detected, regardless of panel density or population type. However, quantitative trait loci (QTL) regions were only found for a few traits including TMCOL Minolta A* and GMCOL Minolta B* (CBs), FMCOL Minolta B*, FMCOL Minolta L*, and ICOL Minolta B* (PBs) and FMCOL Minolta A*, FMCOL Minolta B*, GMCOL Minolta B*, and ICOL Minolta B* (Combined dataset). More QTL regions were identified with WGS (*n* = 58) relative to medium-density marker panels (*n* = 22). Most of the QTL were linked to previously reported QTLs or candidate genes that have been previously reported to be associated with meat quality, pH and pork color; e.g., *VIL1, PRKAG3, TTLL4,* and *SLC11A1, USP37*. *CTDSP1* gene on SSC15 has not been previously associated with meat color traits in pigs. The findings suggest any added value of WGS was only for detecting novel QTL regions when the sample size is sufficiently large as with the Combined dataset in this study. The percentage of phenotypic variance explained by the most significant SNPs also increased with WGS compared with medium-density panels. The results provide additional insights into identification of a number of candidate regions and genes for pork color traits in different pig populations.

## Introduction

Pork color is a key effective indicator for meat quality traits and freshness, since it has been shown that there is a moderate to high association between some of the pork color traits and other meat quality traits such as drip loss (e.g., genetic correlation of 0.55 ± 0.24 and 0.42 ± 0.19 between drip loss and Loin Minolta L* and Loin Minolta A*, respectively) and ultimate pH (genetic correlation of -0.37 ± 0.16) (Miar et al., 2014). Therefore, pork color is an important factor which influences consumer decisions for purchasing pork (Glitsch, 2000). Moreover, Miar et al. (2014) showed that pork color traits had moderate to high heritability, ranging from 0.10 ± 0.05 to 0.38 ± 0.06 (average = 0.25). This shows that in addition to the environmental factors, genetic factors control pork color. Hence, genetic improvement of pork color, which is economically important for the swine industry, is possible in pig breeding programs. Understanding the complex genetic mechanisms underlying pork color traits, which can be done by detection of new genomic regions associated with these traits, is a necessity for the genetic improvement of these traits. Genome-wide association studies (GWAS) using a part of the data^1^ in the current study, have identified several regions associated with pork color traits (Zhang et al., 2015; Yang et al., 2017). Five genomic regions on *Sus scrofa* chromosomes (SSC) 1, 5, 9, 15, 16 and the X chromosome were identified (Zhang et al., 2015). The region on SSC15 spanning 133–134 Mb explained 3.51%–17.06% of genetic variance for five measurements of pH and color (Zhang et al., 2020). Yang et al. (2017) identified 20 genomic regions associated with 18 pork color traits. Three of the genomic regions (on 32–36 Mbp of SSC1 for quadriceps femoris muscle (QFCOL) Minolta A*, 130–134 Mbp of SSC15 for three traits (QFCOL Minolta A* and B*, thawed loin muscle (TMCOL) Minolta B*), and a region on SSC16) associated with three pork color traits identified by Zhang et al. (2015) were also detected by Yang et al. (2017).

To date, most GWAS have used medium-to high-density marker panels to detect the genomic regions associated with carcass and meat quality traits. Use of whole-genome sequence (WGS) is expected to improve identification of associated regions (in terms of both distinct and extended candidate regions and identifying novel genomic regions), because most of the causative variants are expected to be within WGS. The causative SNPs have low MAF (rare variants) and their variance is expected to be captured using WGS. According to simulations, using WGS data for GWAS, the precision of mapping for rare variants increased considerably, which supports the efficiency of WGS in detecting and fine-mapping of low frequency variants simultaneously (Wu et al., 2017). Identification of such variants can increase the utility of genomic selection (GS) for traits such as pork quality by increasing selection accuracy, particularly in multi-population or across population genetic evaluations as used in most commercial pig production which uses crossbreeding and ultimately accelerating genetic gain (Kizilkaya et al., 2014). A disadvantage of using WGS for genetic analyses is the cost of sequencing. Even though the costs of WGS are decreasing, it is still too expensive to sequence at sufficient coverage the thousands of animals required for accurately detecting the genomic regions associated with complex quantitative traits such as pork color traits. A promising alternative is to sequence influential founder animals with the highest genetic contribution to the target population (so-called “reference population”) and to impute the sequence of the remaining animals from low density genotypes (so-called “target population”) (Meuwissen and Goddard, 2010a; b). A cost-effective sequencing alternative to obtain large-scale genomic information is low-pass whole-genome sequence in which 1x coverage or less of a target genome is sequenced. Low-pass sequencing combined with imputation has been proposed as an alternative to genotyping arrays for improving both quantitative trait loci (QTL) detection through a GWAS (Li et al., 2021) and genomic prediction accuracy (Snelling et al., 2020).

Through imputation, based on WGS, the missing variants in the target population can be predicted by use of linkage and segregation analysis. Imputation accuracy is an important factor for more accurate detection of associated regions. Bouwman et al. (2018) assessed the accuracy of imputation from a 70K SNP panel to WGS, from a 660K SNP panel to WGS, and a two-step procedure from 70K to 660K to WGS, using three imputation programs including Beagle 4.1 (Browning et al., 2018), Minimac3 (Das et al., 2016), and FImpute (Sargolzaei et al., 2014). They showed that using a small reference set of 168 sequenced pigs, imputation from 660K was more accurate than imputation from 70K directly to WGS. Their two-step procedure (from 70K to 660K to WGS) resulted in the lowest imputation accuracy. They also showed that Beagle 4.1 outperformed Minimac3. In their study, FImpute performed less well compared with other imputation programs. A useful strategy to reduce imputation error rate is to filter SNPs based on their imputation accuracy prior to analysis.

The use of imputed WGS has been more common in GWAS for pig traits in recent years (Li et al., 2017; Yan et al., 2017; Yan et al., 2018; Van den Berg et al., 2019; Wu et al., 2019; Yang et al., 2021). Van den Berg et al. (2019) showed that using the imputed WGS, the detected QTLs increased with increasing SNP density. They found that compared to 80K and 660K genotypes, using imputed WGS led to the identification of 48.9 and 64.4% more QTL regions, for Landrace and Large White pigs, respectively, and the most significant SNPs in the QTL regions explained a higher proportion of phenotypic variance. Wu et al. (2019) detected 113 and 18 SNPs associated with farrowing interval of different parities in two pig populations using imputed sequence variants. Also, Yan et al. (2017) identified a QTL associated with lumbar number in Sutai pigs using imputed WGS. Nevertheless, to the best of our knowledge, few studies have investigated using imputed WGS for GWAS for meat and carcass quality traits in both purebred and crossbred pigs.

We performed GWAS for 18 meat color traits including Minolta L*, A*, and B* for fat (FCOL), quadriceps femoris muscle (QFCOL), thawed loin muscle (TMCOL), fresh ham gluteus medius (GMCOL), ham iliopsoas muscle (ICOL), and longissimus dorsi muscle on the fresh loin (FMCOL). Analyses were conducted for two datasets: 954^2^ crossbred pigs (CBs) and 891^3^ purebred pigs (PBs). Sequence variants, called across the 60 sequenced pigs, were imputed from a medium-density marker panel (61K for CBs and 50K for PBs) in all genotyped pigs. We applied a single marker association analysis and accounted for polygenic effects through the genomic relationship matrix for each dataset. The main objectives of the study were therefore: 1) to assess the imputation accuracy from 61K CBs and 50K PBs to WGS using a small reference population of 60 sequenced pigs, and 2) to investigate whether the use of WGS detected more associated regions compared with lower density SNP panels. Furthermore, we performed GWAS on combined CBs and PBs to assess whether or not the power of GWAS increased with increasing population size. Finally, we identified potential candidate genes within the associated regions and described the biological roles of the most interesting regions through functional analyses.

## Materials and methods

### Data

#### Phenotypes

This study was performed using the data provided by Hendrix Genetics (Hypor Inc., *Regina*, SK, Canada). Phenotypes of 18 meat color traits were available for 1,037 commercial crossbred pigs (524 female and 513 male CBs, mostly from three-way cross between Duroc boars and Landrace-Yorkshire sows, and 76 were from F1 hybrid sows (Landrace-Yorkshire)). Also, phenotypes of 15 meat color traits were available for 891 purebred Duroc females. The list of the 18 meat color traits and their abbreviations are given in Table 1. Number of individuals in the pedigree were 4,420 and 5,260 for CBs and PBs, respectively. The combined PB and CB pedigree was made by defining the genetic groups in ASReml program V4.0 (Gilmour et al., 2015), as the animals from PBs and CBs were considered to belong to different genetic groups. Thus, the combined pedigree comprised 6,419 individuals including the genetic groups. The details on how the pork color phenotypes were measured in the six locations of the pork have been described in Yang et al. (2017).

**TABLE 1:** List of pork color traits and their abbreviations.

Number	Trait abbreviation	Trait description
1	FCOLA	Fat Minolta A^*^
2	FCOLB	Fat Minolta B^*^
3	FCOLL	Fat Minolta L^*^
4	QFCOLA	Quadriceps femoris muscle Minolta A^*^
5	QFCOLB	Quadriceps femoris muscle Minolta B^*^
6	QFCOLL	Quadriceps femoris muscle Minolta L^*^
7	FMCOLA	Fresh marbling color A^*^ - longissimus dorsi
8	FMCOLB	Fresh marbling color B^*^ - longissimus dorsi
9	FMCOLL	Fresh marbling color L^*^ - longissimus dorsi
10	TMCOLA	Thawed loin muscle Minolta A^*^
11	TMCOLB	Thawed loin muscle Minolta B^*^
12	TMCOLL	Thawed loin muscle Minolta L^*^
13	GMCOLA	Ham gluteus medius Minolta A^*^
14	GMCOLB	Ham gluteus medius Minolta B^*^
15	GMCOLL	Ham gluteus medius Minolta L^*^
16	ICOLA	Ham iliopsoas Minolta A^*^
17	ICOLB	Ham iliopsoas Minolta B^*^
18	ICOLL	Ham iliopsoas Minolta L^*^

### Genotypes

Of the 1,037 crossbred individuals that had phenotypic records, 941-954 individuals (depending on the trait) had both phenotypes and genotypes with a custom 61K (61,565 SNPs)^4^ Illumina SNP panel (Table 2). Genotyping of CBs was performed by Delta Genomics (Edmonton, AB, Canada) using Illumina PorcineSNP60 V2 Genotyping Beadchip according to the Illumina Infinium Assay (Illumina, Inc., San Diego, CA, United States). Of the 891 purebred Duroc females that had phenotypic records, 873-891 individuals (depending on the trait) had both phenotypes and genotypes with a custom 50K (50,703 SNPs) Illumina SNP panel (Table 2). Genotyping of purebred pigs was performed by Neogen Corporation - GeneSeek operations (Lincoln, Nebraska, NE, United States). Based on the “proportion of genetic diversity” approach (Druet et al., 2014), 60 Duroc boars were identified as key ancestors of the PB population and DNA of these boars was used for sequencing. Moreover, for 17 of the 891 purebred Duroc sows, genotypes from the 660K SNP panel including 659,692 SNPs were available. We used this set of individuals to assess potential increase in imputation accuracy when using a two-step procedure. The two-step procedure was from 50K to 660K to WGS, while in the one-step approach the imputation was conducted from 50K to WGS directly.

**TABLE 2:** The descriptive statistics for 18 pork color traits: number of animals per trait (N), means, SD, minimum (Min.), and maximum (Max.) values for different datasets (CB, PB, and Combined dataset).

	CB					PB					Combined dataset				
Trait	N	Mean	SD	Min	Max	N	Mean	SD	Min	Max	N	Mean	SD	Min	Max
FCOLA	941^a^	3.71	1.13	0.70	7.90	873	2.84	1.34	-0.40	7.30	1844	3.38	1.52	−0.40	19.20
FCOLB	953	18.31	1.48	11.70	24.40	885	10.84	1.75	6.00	17.20	1844	14.70	4.08	3.80	24.40
FCOLL	953	75.29	1.63	66.60	79.80	891	78.96	2.40	64.00	84.60	1844	77.06	2.74	64.00	84.60
QFCOLA	953	4.82	1.60	0.70	11.30	881	2.39	1.37	-1.00	6.70	1844	3.68	1.96	-1.00	13.40
QFCOLB	953	13.61	1.57	9.60	18.70	885	8.27	1.35	4.70	12.10	1844	11.04	3.04	4.70	18.70
QFCOLL	953	49.42	3.46	39.10	62.10	881	53.23	3.37	42.10	65.50	1844	51.27	4.04	36.50	68.90
FMCOLA	953	6.07	1.47	2.00	11.48	891	4.58	1.12	1.08	8.75	1844	5.35	1.51	1.08	11.48
FMCOLB	953	14.91	1.69	10.38	21.90	891	9.38	1.24	5.95	13.90	1844	12.24	3.14	5.95	21.90
FMCOLL	953	48.46	2.64	39.88	60.50	891	48.15	2.53	41.43	55.73	1844	48.31	2.59	39.88	60.50
TMCOLA	950	7.65	1.19	3.39	11.39	-	-	-	-	-	-	-	-	-	-
TMCOLB	950	2.70	1.29	-1.54	7.48	-	-	-	-	-	-	-	-	-	-
TMCOLL	950	44.26	3.11	31.99	55.88	-	-	-	-	-	-	-	-	-	-
GMCOLA	953	6.74	1.20	2.40	10.70	891	5.46	1.27	1.20	9.60	1844	6.12	1.39	1.20	10.70
GMCOLB	953	13.63	1.11	9.60	17.30	891	8.91	1.13	5.40	12.70	1844	11.35	2.61	5.40	17.30
GMCOLL	953	45.31	2.45	38.00	54.20	891	47.50	2.65	39.30	57.20	1844	46.37	2.77	38.00	57.20
ICOLA	953	19.30	1.73	12.00	24.10	891	15.97	2.17	8.80	23.00	1844	17.69	2.59	1.60	24.10
ICOLB	953	13.61	1.57	9.60	18.70	891	11.33	1.50	5.50	15.80	1844	12.51	1.91	5.50	18.70
ICOLL	953	42.54	2.86	35.10	51.80	891	44.04	3.08	34.70	55.60	1844	43.26	3.06	34.70	55.60

a The total number of PBs, with both phenotypes and genotypes were 891. However, for these traits, there were extreme phenotypic records which were removed in the analyses to check if the GWAS, results would improve. Due to little changes in GWAS, results for PBs, those removed individuals were added to the analyses of combined CBs, and PBs. CB, crossbred; PB, purebred; N, number of animals; SD, standard deviation; Min, minimum; Max, maximum.

### Collection of deoxyribonucleic acid samples, deoxyribonucleic acid extraction, library preparation and next-generation sequencing

Genomic DNA extraction from blood and tissue was carried out using the Qiagen DNeasy extraction protocol (Qiagen, Mississauga, ON) by Delta genomics. Extracted DNA was quantified using the Qubit dsDNA HS Assay (Life Technologies, Burlington, ON). 100ng to 1ug of gDNA was sheared using the Covaris S2 focused sonicator (Covaris Inc.) to achieve a fragment size ranging from 300 to 400bp. Sheared DNA fragments were used for library preparation according to respective library preparation protocol that were compatible with Illumina next generation sequencing platform. Quality check and library preparations were done by NEOGEN Canada (Edmonton, AB, Canada). Sequencing was done by McGill University and Génome Québec Innovation Centre (Montréal, Québec, Canada). Libraries were normalized and pooled and then denatured in 0.05N NaOH and neutralized using HT1 buffer. ExAMP was added to the mix following the manufacturer’s instructions. The pool was loaded at 200pM on a Illumina cBot and the flowcell was run on a HiSeq X for 2 × 151 cycles (paired-end mode). A phiX library was used as a control and mixed with libraries at 1% level. The Illumina HiSeq Control Software was HCS HD 3.4.0.38, and the real-time analysis program was RTA v. 2.7.7. Program bcl2fastq2 v2.20 was then used to de-multiplex samples and generate fastq reads.

### Sequence depth, read trimming, alignment, and variant calling

Sequence reads trimming and adapter clipping was performed using Trimmomatic algorithm 0.38 (Bolger et al., 2014). The average sequence coverage was computed using *depth* in VCFTOOLS (Danecek et al., 2011) and was 21.75 across the 60 sequenced animals (Supplementary Table S1). Sequence reads alignment was conducted using the current pig reference genome (*Sus scrofa* 11.1 (https://uswest.ensembl.org/Sus_scrofa/Info/Index), www.ensembl.org/biomart/martview) with BWA *mem* (BWA 0.7.17) using the default parameters (Li and Durbin, 2009). The alignment SAM files were converted to BAM format using Samtools-0.1.19 (Li et al., 2009). Next, BAM files were sorted and indexed by Samtools 1.8 (Li et al., 2009). Potential PCR duplicates were removed by tool *MarkDuplicates* from Picard v2.18.2 (http://broadinstitute.github.io/picard/). Variants (SNPs and insertion-deletions (INDELs)) were called using GenomeAnalysisToolKit-3.8-1-0 (GATK) (McKenna et al., 2010). Tool *HaplotypeCaller* was used for variant calling. Default parameter settings of *HaplotypeCaller* were used for variant calling, except for the following parameters: minimum base quality required to consider a base for calling equal to 20 and the minimum phred-scaled confidence threshold for variant calling equal to 20. Base quality recalibration was performed according to GATK best practices guidelines using tools *BaseRecalibrator* and *PrintReads* (McKenna et al., 2010; van der Auwera et al., 2013). Finally, BAM files were pooled for variant calling. In the 60 Duroc males, the total numbers of SNPs and INDELs called were more than 19 and more than five million, respectively.

### Quality control of called sequenced variants

During variant calling, the variants were filtered using parameters recommended by GATK Best Practices (DePristo et al., 2011). Some other filters were applied to choose sequencing variants for GWAS analyses. Due to the complexity of imputation for INDELS, we only used SNPs as variants in this study. The following filters were applied to SNPs before subsequent analyses. A SNP was excluded with: the strand bias *p*-value < 0.01 calculated with Fischer’s exact test, two or more alternative alleles, a MAF <0.025, missing observation of the alternative allele on either the forward or reverse reads, being located within 4 bp of each other, being located within 5 bp of an INDEL, a mapping quality (MQ) score of <40, a phred score <20, a read depth (DP) of less than 10% of median or more than median plus 3 standard deviation of read depth, a quality depth (QD) < 5. We also removed sex chromosomes. After filtering, 11, 946, 148 SNPs on autosomes (SSC1 to SSC18) remained for the 60 animals across the whole-genome (Table 3).

**TABLE 3:** Total number of SNPs, chromosome length, and average imputation accuracy (allelic DR^2^) per chromosome after filtrations, and before and after imputation filtration criteria (allelic DR^2^) in crossbreds (CBs) and purebreds (PBs).

		CB				PB			
		Before filtering on allelic DR^2^		After filtering allelic DR^2^ > 0.8 on CBs		Before filtering on allelic DR^2^		After filtering allelic DR^2^ > 0.8 on PBs	
Chromosome	Length (Mb)	Total number of SNPs	Mean allelic DR^2^	Total number of SNPs	Mean allelic DR^2^	Total number of SNPs	Mean allelic DR^2^	Total number of SNPs	Mean allelic DR^2^
SSC1	274	945,428	0.83	641,461	0.92	945,428	0.91	831,268	0.98
SSC2	152	791,977	0.81	509,047	0.92	791,977	0.88	668,474	0.97
SSC3	133	660,517	0.80	402,300	0.91	660,517	0.90	572,923	0.97
SSC4	131	740,467	0.84	517,636	0.92	740,467	0.92	669,685	0.97
SSC5	105	544,278	0.77	285,211	0.90	544,278	0.89	466,844	0.96
SSC6	171	824,770	0.79	475,917	0.91	824,770	0.89	696,109	0.96
SSC7	122	679,812	0.81	432,486	0.91	679,812	0.91	596,197	0.97
SSC8	139	866,293	0.81	534,659	0.91	866,293	0.92	774,027	0.97
SSC9	140	728,874	0.79	435,857	0.91	728,874	0.90	626,371	0.97
SSC10	69	579,468	0.79	341,971	0.90	579,468	0.88	483,451	0.95
SSC11	79	529,008	0.79	311,109	0.91	529,008	0.90	465,485	0.96
SSC12	62	429,775	0.79	244,130	0.90	429,775	0.88	359,175	0.95
SSC13	208	872,065	0.82	564,990	0.91	872,065	0.92	781,689	0.97
SSC14	142	755,463	0.81	480,616	0.92	755,463	0.91	660,825	0.97
SSC15	140	708,954	0.82	463,330	0.91	708,954	0.92	636,278	0.97
SSC16	80	523,910	0.80	320,423	0.91	523,910	0.91	465,914	0.96
SSC17	63	459,174	0.77	254,861	0.91	459,174	0.89	384,099	0.96
SSC18	56	305,915	0.79	177,266	0.92	305,915	0.91	270,808	0.97
Total/Average	126	11,946,148	0.80	7,393,270	0.91	11,946,148	0.90	10,409,622	0.97

CB, crossbred; PB, purebred; SNP, single nucleotide polymorphism; Mb, megabyte; SSC, *sus scrofa.*

### Quality control of 50K, 61K, and 660K SNP panel

Quality control of the 50K (for 891 PBs), 61K (for 954 CBs) and 660K (for 17 PBs) were as follows: SNPs were excluded if they were duplicated, if they had a MAF <0.01. Furthermore, SNPs with genotype call rate <0.95 and SNPs with unknown map positions were removed. The quality control of genotypes was done for each trait separately, because the number of animals with both genotypes and phenotypes differ among the pork color traits. The numbers of SNPs after these exclusions are indicated in Table 4.

**TABLE 4:** Number of individuals and SNPs used for GWAS after quality control for different datasets (61K genotypes of CBs, 50K genotypes of PBs, and Combined dataset).

	CB			PB			Combined dataset		
Trait	61K	N	Imputed WGS	50K	N	Imputed WGS	61K + 50K	N	Imputed WGS
FCOLA	44,098	941^a^	7,376,594	35,775	873^a^	10,094,644	29,349	1,844	10,331,074
FCOLB	44,068	953	7,376,594	35,799	885^a^	10,090,482	29,349	1,844	10,331,074
FCOLL	44,068	953	7,377,298	35,782	879^a^	10,096,911	29,349	1,844	10,331,074
QFCOLA	44,070	953	7,376,594	35,801	882^a^	10,095,407	29,349	1,844	10,331,074
QFCOLB	44,068	953	7,376,594	35,809	885^a^	10,097,161	29,349	1,844	10,331,074
QFCOLL	44,068	953	7,376,594	35,809	881^a^	10,097,982	29,349	1,844	10,331,074
FMCOLA	44,070	953	7,376,594	35,809	891	10,099,578	29,349	1,844	10,331,074
FMCOLB	44,070	953	7,376,594	35,809	891	10,099,578	29,349	1,844	10,331,074
FMCOLL	44,070	953	7,376,594	35,809	891	10,099,578	29,349	1,844	10,331,074
TMCOLA	44,103	950	7,377,387	-	-	-	-	-	-
TMCOLB	44,103	950	7,377,387	-	-	-	-	-	-
TMCOLL	44,103	950	7,377,387	-	-	-	-	-	-
GMCOLA	44,070	953	7,376,594	35,809	891	10,099,578	29,349	1,844	10,331,074
GMCOLB	44,070	953	7,376,594	35,809	891	10,099,578	29,349	1,844	10,331,074
GMCOLL	44,070	953	7,376,594	35,809	891	10,099,578	29,349	1,844	10,331,074
ICOLA	44,070	953	7,376,594	35,809	891	10,099,416	29,349	1,844	10,331,074
ICOLB	44,070	953	7,376,594	35,809	891	10,099,578	29,349	1,844	10,331,074
ICOLL	44,070	953	7,376,594	35,809	891	10,099,578	29,349	1,844	10,331,074
Min	44,068	950	7,376,594	35,775	891	10,090,482	29,349	1,844	10,331,074
Max	44,103	954	7,377,387	35,809	891	10,099,578	29,349	1,844	10,331,074

a The total number of PBs, with both phenotypes and genotypes were 891. However, for these traits, there were extreme phenotypic records which were removed in the analyses to check if the GWAS, results would improve. Due to little changes in GWAS, results for PBs, those removed individuals were added to the analyses of combined CBs, and PBs. CB, crossbred; PB, purebred; WGS, whole-genome sequence; N, number of animals.

### Imputation to whole-genome sequence

Beagle V5.0 (Browning et al., 2018) was used for imputation of 61K genotypes of CBs, 50K genotypes of PBs, and 660K genotypes of 17 purebred Duroc sows to the WGS (60 sequenced pigs). Default parameter settings of Beagle V5.0 were used, except for number of iterations for genotype phasing (default value was 12, but we used 25), and for effective population size (default value was 1,000,000 which is appropriate for a large population such as the human population, but we used 100 for our pig populations which helps with accurate imputation of small populations (Browning et al., 2018)). Pedigree information was not used for imputation.

Evaluation of imputation accuracy is needed particularly for SNPs with low minor allele frequency (MAF) which are abundant in WGS. Evaluation of imputation accuracy was done in two ways. The first measure of imputation accuracy per SNP was obtained from the allelic DR^2^ generated by Beagle, which is defined as the squared correlation between the expected dose (i.e., P (AB) + 2*P(BB)) and the true dose (Browning et al., 2018). Second, we were interested in imputation accuracy per pig (animal-specific imputation accuracy). True and imputed genotypes are needed to evaluate animal-specific imputation accuracy. Of the 60 sequenced pigs, 61K genotypes were available for 55 individuals which were used for assessing the animal-specific imputation accuracy using leave-one-out cross validation. Imputation accuracy was defined as the correlation between true and the most likely imputed genotypes. The leave-one-out cross validation analyses were performed using both Beagle V5.0 and FImpute (Sargolzaei et al., 2014) to compare the performance of the two programs. Due to large computation time, animal-specific imputation accuracy was assessed with the data for SSC18 only. For FImpute, the default values on all parameters were used, except for the error rate threshold to find progeny-parent mismatches, shrink factor for sliding windows, and amount of overlap for sliding windows. The values used for progeny-parent mismatches, shrink factor, and amount of overlap for sliding windows were 0.03, 0.15, and 0.65, respectively.

To assess whether a two-step imputation strategy would improve imputation accuracy compared with a one-step imputation strategy, particularly for low MAF SNPs (Kreiner-Møller et al., 2014; Lent et al., 2016; Bouwman et al., 2018), we performed imputation, using Beagle V5.0 only, from 50K SNP panel to WGS with 60 Duroc boars (one-step imputation strategy) and from 50K SNP panel to 660K SNP panel to WGS with 60 Duroc boars (two-step imputation strategy).

### Quality control of imputed genotypes

Imputed genotypes were filtered based on the imputation reliability (allelic DR^2^) produced by Beagle (Table 4). The chosen cut-off threshold for filtrations of allelic DR^2^ was 0.8. The reason for adapting a cut-off threshold of 0.8 was to achieve a balance between the average imputation reliability and the number of excluded SNPs. Consequently, of the 11,946,148 SNPs used for imputation, after exclusion of SNPs with imputation reliability less than 0.8, 7,393,270 and 10, 409, 622 SNPs remained for further analyses for CBs and PBs, respectively (Table 3).

### Variance component estimates

Variance components, additive genetic variance ( $\sigma_{A}^{2}$ ) and residual variance ( $\sigma_{E}^{2}$ ), were estimated *via* the restricted maximum likelihood (REML) using ASReml program V4.0 (Gilmour et al., 2015) using a best linear unbiased prediction (BLUP) animal model as follows:

$$y=1\mu +Xb+Z_{a}a+e$$ (1)

where y is the vector of phenotypic records, 1 is a vector of ones, **μ** is overall mean of phenotypic records, b is a vector of fixed class effects (the significant fixed effects for each trait is given in Table 5), **X** is a design matrix corresponding to the fixed effects, **a** is a vector of breeding values considered as random effects, $Z_{a}$ is an incidence matrix that related phenotypic records to breeding values, and **e** is a vector of random residual effects. It is assumed that $a\;∼\;N\left(0,A\sigma_{a}^{2}\right)$ and $e\;∼\;N\left(0,I\sigma_{e}^{2}\right)$ where $\sigma_{a}^{2}$ and $\sigma_{e}^{2}$ are the additive genetic and residual variances, respectively, and A is the numerator relationship matrix based on pedigree. Moreover, a narrow-sense heritability ( $h^{2}$ ) was calculated as the division of the additive genetic variance by the total phenotypic variance as shown in Table 6. Standard errors of the variance components were also estimated by ASReml.

**TABLE 5:** Significance of the fixed effects (sex, slaughter date, room, pen, birth year-month, and population) included in the mixed model for different datasets (CB, PB, and Combined dataset) for the pork color traits.

	CB					PB				Combined dataset						
Trait	Sex	Slaughter date	Room	Pen	Birth year-month	Slaughter date	Room	Pen	Birth year-month	Sex	Slaughter date	Room	Pen	Birth year-month	population	
FCOLA	**	***	NS	**	NS	*	NS	NS	*	***	***	NS	NS	NS	**	
FCOLB	***	***	NS	NS	NS	***	NS	NS	***	***	***	NS	NS	NS	***	
FCOLL	***	NS	NS	***	***	NS	*	NS	***	*	***	NS	NS	NS	***	
QFCOLA	NS	NS	NS	NS	***	NS	NS	NS	***	NS	***	NS	NS	NS	***	
QFCOLB	NS	***	NS	NS	***	NS	***	**	***	NS	***	NS	NS	NS	***	
QFCOLL	NS	**	NS	NS	NS	NS	NS	*	NS	NS	***	NS	*	NS	***	
FMCOLA	***	NS	**	NS	***	NS	NS	NS	***	***	***	NS	NS	NS	***	
FMCOLB	*	NS	NS	NS	***	***	NS	NS	***	**	***	NS	NS	NS	***	
FMCOLL	NS	***	NS	NS	*	NS	NS	NS	***	NS	***	NS	NS	**	NS	
TMCOLA	***	***	NS	NS	***	-	-	-	-	-	-	-	-	-	-	
TMCOLB	*	***	NS	NS	***	-	-	-	-	-	-	-	-	-	-	
TMCOLL	NS	***	NS	NS	***	-	-	-	-	-	-	-	-	-	-	
GMCOLA	NS	***	*	NS	NS	***	NS	NS	***	***	***	**	NS	NS	*	
GMCOLB	NS	***	NS	NS	***	***	NS	NS	NS	*	***	NS	NS	NS	***	
GMCOLL	NS	***	NS	*	*	***	NS	NS	NS	*	***	NS	NS	NS	***	
ICOLA	NS	***	NS	**	***	***	NS	NS	***	*	***	NS	NS	NS	***	
ICOLB	NS	***	NS	NS	NS	***	NS	NS	NS	NS	***	NS	NS	NS	***	
ICOLL	NS	***	NS	NS	NS	NS	***	NS	***	***	***	*	NS	NS	***	

CB, crossbred; PB, purebred; NS: non-significant. ***p < 0.01; **p < 0.05; *p < 0.1.

**TABLE 6:** Variance component estimates (additive and residual variances), and estimates of total heritability for 18 pork color traits for different datasets (CB, PB, and Combined dataset).

	CB			PB			Combined dataset					
Trait	$\sigma_{A}^{2}$ (se)	$\sigma_{E}^{2}$ (se)	$h^{2}$ (se)	$\sigma_{A}^{2}$ (se)	$\sigma_{E}^{2}$ (se)	$h^{2}$ (se)	$\sigma_{A}^{2}$ (se)		$\sigma_{E}^{2}$ (se)		$h^{2}$ (se)	
							CB	PB	CB	PB	CB	PB
FCOLA	0.37 (0.12)	1.07 (0.10)	0.26 (0.08)	0.33 (0.18)	2.39 (0.19)	0.12 (0.06)	0.33 (0.11)	0.32 (0.17)	1.03 (0.11)	2.15 (0.22)	0.24 (0.08)	0.13 (0.07)
FCOLB	0.41 (0.10)	0.64 (0.07)	0.39 (0.08)	0.48 (0.18)	1.61 (0.16)	0.23 (0.08)	0.39 (0.10)	0.39 (0.15)	0.57 (0.08)	1.46 (0.17)	0.40 (0.09)	0.21 (0.08)
FCOLL	0.54 (0.30)	1.52 (0.19)	0.26 (0.13)	0.56 (0.35)	4.84 (0.37)	0.10 (0.06)	0.43 (0.15)	0.66 (0.36)	1.4 (0.15)	4.23 (0.42)	0.23 (0.08)	0.13 (0.07)
QFCOLA	1.00 (0.23)	1.31 (0.16)	0.43 (0.08)	0.72 (0.22)	1.55 (0.18)	0.32 (0.09)	0.85 (0.21)	0.77 (0.22)	1.37 (0.17)	1.51 (0.26)	0.38 (0.08)	0.34 (0.09)
QFCOLB	0.46 (0.16)	1.55 (0.13)	0.23 (0.07)	0.12 (0.10)	1.59 (0.12)	0.07 (0.06)	0.47 (0.15)	0.10 (0.09)	1.56 (0.16)	1.56 (0.21)	0.23 (0.07)	0.06 (0.06)
QFCOLL	4.35 (1.04)	7.68 (0.79)	0.36 (0.08)	1.19 (0.81)	12.09 (0.91)	0.09 (0.06)	5.07 (1.13)	1.30 (0.83)	8.30 (0.98)	14.32 (1.34)	0.38 (0.07)	0.08 (0.05)
FMCOLA	0.86 (0.19)	0.94 (0.13)	0.48 (0.09)	0.46 (0.13)	0.77 (0.10)	0.38 (0.09)	0.67 (0.17)	0.44 (0.12)	1.06 (0.14)	0.81 (0.17)	0.39 (0.08)	0.35 (0.10)
FMCOLB	0.52 (0.15)	1.14 (0.11)	0.31 (0.08)	0.20 (0.09)	0.92 (0.09)	0.18 (0.08)	0.46 (0.15)	0.16 (0.08)	1.18 (0.13)	1.07 (0.16)	0.28 (0.08)	0.13 (0.07)
FMCOLL	2.73 (0.63)	2.70 (0.41)	0.50 (0.09)	1.06 (0.45)	4.30 (0.41)	0.20 (0.08)	3.65 (0.75)	1.08 (0.46)	2.47 (0.52)	4.35 (0.61)	0.60 (0.09)	0.20 (0.08)
TMCOLA	0.62 (0.13)	0.48 (0.08)	0.57 (0.09)	-	-	-	-	-	-	-	-	-
TMCOLB	0.34 (0.09)	0.72 (0.07)	0.32 (0.08)	-	-	-	-	-	-	-	-	-
TMCOLL	1.89 (0.53)	4.28 (0.41)	0.31 (0.08)	-	-	-	-	-	-	-	-	-
GMCOLA	0.63 (0.14)	0.72 (0.09)	0.47 (0.08)	0.67 (0.17)	0.88 (0.13)	0.43 (0.10)	0.61 (0.14)	0.58 (0.16)	0.77 (0.11)	0.99 (0.17)	0.44 (0.08)	0.37 (0.10)
GMCOLB	0.20 (0.07)	0.78 (0.06)	0.20 (0.06)	0.29 (0.09)	0.69 (0.08)	0.29 (0.09)	0.17 (0.06)	0.29 (0.09)	0.85 (0.07)	0.86 (0.12)	0.17 (0.06)	0.25 (0.08)
GMCOLL	1.53 (0.44)	3.98 (0.36)	0.28 (0.07)	2.00 (0.62)	4.43 (0.51)	0.31 (0.09)	1.47 (0.42)	2.08 (0.63)	4.16 (0.42)	4.75 (0.71)	0.26 (0.07)	0.31 (0.09)
ICOLA	0.79 (0.21)	1.77 (0.16)	0.31 (0.07)	0.95 (0.38)	3.40 (0.34)	0.22 (0.08)	0.57 (0.18)	1.07 (0.39)	1.71 (0.17)	3.01 (0.41)	0.25 (0.07)	0.26 (0.09)
ICOLB	0.44 (0.15)	1.55 (0.13)	0.22 (0.07)	0.19 (0.12)	1.62 (0.12)	0.10 (0.06)	0.45 (0.15)	0.26 (0.14)	1.57 (0.15)	1.62 (0.22)	0.22 (0.07)	0.14 (0.07)
ICOLL	2.61 (0.62)	3.53 (0.43)	0.43 (0.08)	1.29 (0.61)	6.81 (0.60)	0.16 (0.07)	2.37 (0.60)	1.11 (0.57)	3.31 (0.47)	6.16 (0.76)	0.42 (0.09)	0.15 (0.08)

CB, crossbred; PB, purebred; $\sigma_{A}^{2}$ , additive genetic variance; $\sigma_{E}^{2}$ , residual variance; $h^{2}$ , narrow-sense heritability; se: standard error.

When the CBs and PBs were combined for variance component estimations, the heterogeneous genetic and residual variances were fitted in the model. Since CBs contained both males and females individuals, while PBs contained only female individuals, first an animal model was fitted to check the difference between the residual variances in CB and PBs as well as the difference between the residual variances between the male and female individuals. For all traits, the residual variances were different between the two populations as well as between the two sexes. Then, the first model was expanded to check if there was a difference between the genetic variances between the two populations (CBs *versus* PBs).

For parameter estimation, the data size presented in Table 2 was used, which ranges from 941 (FCOLA) to 954 (QFCOLB) for CBs, from 873 (FCOLA) to 891 for most of the traits. For Combined dataset, the total number of individuals was 1,844 for all traits. Relevant fixed effects fitted in the mixed model analysis for the 18 color traits are in Table 5.

### Genome-wide association analyses

The model used for GWAS was a single-marker mixed linear association model (MLMA, mixed linear model based association analysis) implemented in GCTA version 1.92.1beta6 (Yang et al., 2011; Yang et al., 2014). The statistical model was as follows:

$$\hat{y}=1\mu +Zu+g+e$$ (2)

Where $\hat{y}$ was the vector of phenotypic records corrected for fixed effects (only significant fixed effects was used for correcting each trait, See Table 5). u was the additive effect (fixed effect) of the candidate SNP to be tested for association, Z was a vector containing the SNP genotype indicator variable coded as 0 (AA), 1 (AB), and 2 (BB). g was a vector of random polygenetic effects, and **e** was a vector of random residual effects. It was assumed that $g\;∼\;N\left(0,G\sigma_{g}^{2}\right)$ and $e\;∼\;N\left(0,I\sigma_{e}^{2}\right)$ , where $\sigma_{g}^{2}$ and $\sigma_{e}^{2}$ were the genetic and residual variances, respectively. G was the genomic relationship matrix based on genotypes, constructed using GCTA software tool (Yang et al., 2011). GWAS was done using both medium-density panels and WGS data.

### Significance testing

The significance threshold of SNP effects was assessed by using a false discovery rate (FDR) of 0.1 (Benjamini and Hochberg, 1995). Such threshold is needed to reduce the number of unacceptable false positives due to multiple testing. To account for population structure, the GWAS *p*-values for each trait were corrected for their corresponding genomic inflation factor (here called lambda) (Yang et al., 2011). Lambda was used for evaluating the bias. Lambda values for each data panel (medium-density and WGS) were computed as the median of the observed chi squared test statistics divided by the expected median of the corresponding chi squared distribution assuming 1 degree of freedom. *p*-values were used to compute the chi square test statistics. Moreover, quantile-quantile (qq) plot for each trait was used to evaluate the inflation of *p*-values by comparing the genome wide distribution of -log10 of the *p*-values with the expected median of the corresponding chi squared distribution assuming a degree of freedom of one.

### Linkage disequilibrium decay

**FIGURE 1:**
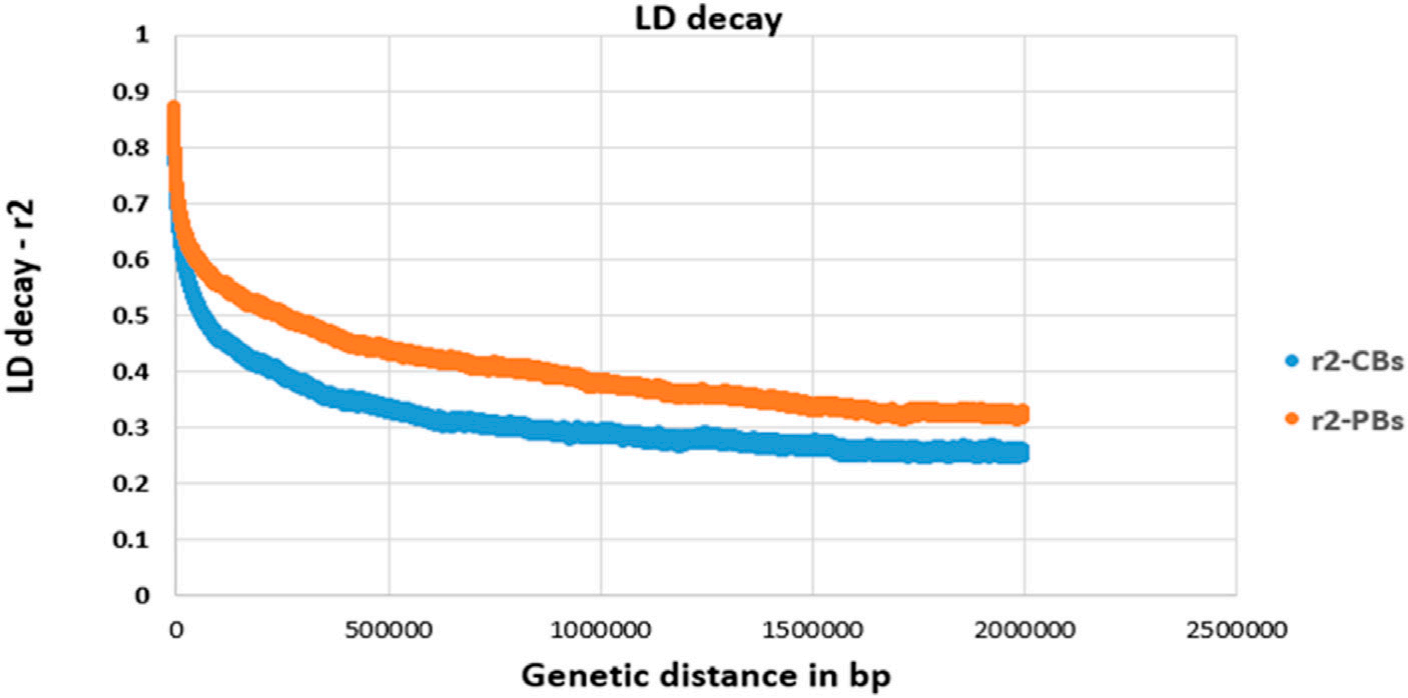
Linkage disequilibrium (LD, *r* ^2^) decay for SSC1 of CBs and PBs as a function of inter-SNP distance. Physical (genetic) distance is measured in base pair (bp).

LD decay pattern between pairwise SNPs (imputed sequence) was evaluated for both CBs and PBs. The pairwise LD values (*r* ^2^, defined as the correlation between alleles of two SNPs harbored at different loci (Hill & Robertson, 1968) between SNP pairs were computed for SNPs located within 2000 Kb windows and shorter (Figure 1). Due to large computation time, LD analyses were only done for SSC1.

### Quantitative trait loci definition

For all traits, we defined the quantitative trait loci (QTL) regions according to the definition described by van den Berg et al. (2019) as follows. First, the SNPs on each chromosome were ranked based on their -log10 *p*-values. Secondly, starting with the SNPs with the largest -log10 *p*-value, all significant SNPs that exceeded the FDR of 0.1 and surrounding SNPs within a 0.5 Mb region to the left and right of the SNP were assigned to that QTL region. These two steps were repeated until all significant SNPs were assigned to a QTL region. A distance of 0.5 Mb was chosen as the average LD of commercial pig lines decreases to less than 0.3 (Figure 1) when the SNPs are more than 0.5 Mb apart.

### Variance explained by significant variants

The percentage of phenotypic variance explained by each SNP was estimated as: $\frac{2*p*q*a^{2}}{phenotypic\;variance}$ , where *p* and *q* are the allele frequencies of major and minor alleles, and *a* is the estimated allele substitution effect. It should be noted that for the Combined dataset, the average of phenotype variance of crossbreds and purebreds was used for computation of variance explained.

### Post-genome-wide association studies analyses

After GWAS, candidate gene identification and functional annotation for the significant SNPs were obtained using Ensemble annotation of *Sus scrofa* 11.1 (https://www.ensembl.org/info/data/biomart/). Genomic regions associated with the pork color traits were identified using a 1 Mb window (up- and down-stream of significant peak). The ClueGo plug-in (Bindea et al., 2009) and Cytoscape program (Shannon et al., 2003) were used to group and visualize the genes according to the biological processes in which they are involved in. The ClueGO plug-in uses both Gene Ontology (GO) terms and KEGG/BioCarta pathways to develop a GO/pathway network. Furthermore, ClueGO calculates enrichment and depletion tests for groups of genes based on the hypergeometric distribution and corrects the *p*-values for multiple testing. The *Sus scrofa* database (http://ftp.ensembl.org/pub/current_fasta/sus_scrofa/dna/) was used in pathway and biological processes investigation. We selected the 5^th^ to the 10^th^ levels of the GO hierarchy and a kappa score of 0.4 (Bindea et al., 2009). When no biological functions or pathways were found, these parameters were relaxed to be less stringent.

## Results

Total number of pigs used for GWAS, and the descriptive statistics for 18 pork color traits including the minimum, maximum, mean and standard deviation of traits for different datasets (CB, PB and combined CBs and PBs^5^) are in Table 2. Because of the quality control during and after variant calling on WGS, not all SNPs on the 61K, 660K, and 50K SNP panels were present in the WGS, i.e., for the CBs, 26,585 SNPs of the 61K SNPs and 430,404 SNPs of the 660K SNPs were present, and for the PBs, 34,733 SNPs of the 50K SNPs were present in the WGS.

### Population structure

Supplementary Figure S1 demonstrates (Supplementary Figure S1) population structure among the CBs (*n* = 954) and PBs (*n* = 891) populations, which was computed in Plink using the principal component analysis (PCA) procedure. The common SNPs between 61K and 50K (∼30K) were used for plotting. The blue color shows the PB animals and the red color shows CB pigs. The CB individuals are dispersed across the plot.

### Minor allele frequency distribution

The distribution of MAF from the 61K and 50K SNP panels were uniform, whereas the distribution of MAF from WGS was U-shaped with a substantial proportion of SNPs with small MAF values (approximately 19% of SNPs had a MAF lower than 0.025) (Supplementary Figure S2A). MAF distribution of sequence SNPs used for downstream analyses, after excluding the MAF <0.025, is given in Supplementary Figure S2B. Average MAF across the 28 autosomes before excluding MAF <0.025 was 0.28. After filtration of MAF with 0.025 cut-off threshold, the average MAF was 0.33.

### Evaluation of accuracy of imputation

The average allelic DR^2^ from the 61K and 50K SNP panels to sequence imputation before any filtration was 0.80 and 0.90 across all chromosomes, for CBs and PBs, respectively (Table 3). After filtration of allelic DR^2^ < 0.8, the average allelic DR^2^ from the 61K and 50K SNP panels to sequence imputation across all chromosomes was 0.91 for CBs and 0.97 for PBs (Table 3). The number of SNPs before and after allelic DR^2^ filtration is given in Table 3. Beagle DR^2^ varied between the CBs and PBs and also among the 18 chromosomes. For CBs, the smallest and largest Beagle DR^2^ were obtained for SSC5 (0.77) and SSC4 (0.84), respectively. For PBs, the smallest Beagle DR^2^ were obtained for SSC2 and SSC12 (0.88) and the largest Beagle DR^2^ were obtained for SSC4, 8, 13, and 15 (0.92). Across all chromosomes, the average allelic DR^2^ was larger for PBs than CBs.

**FIGURE 2:**
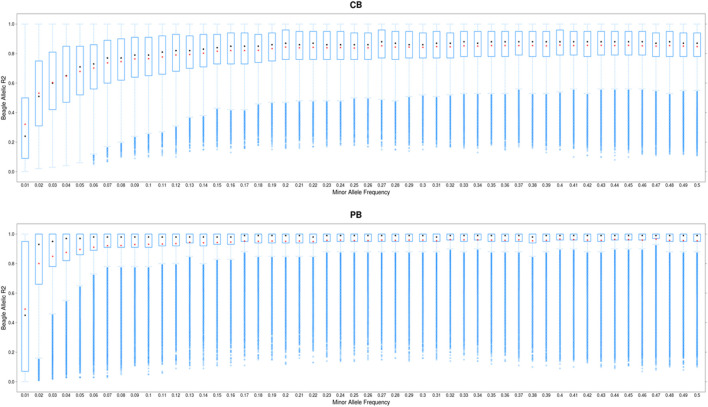
Boxplot showing the imputation accuracy (allelic DR^2^) to whole-genome sequence (WGS) *versus* minor allele frequency (MAF) for CBs and PBs. The *x*-axis represents different classes of MAF (ranging from 0.01 to 0.5, with the steps of 0.01), and *y*-axis shows the imputation accuracies. The red and black dots are the mean and median of imputation accuracies across individuals in each MAF class.

The distribution of allelic DR^2^ against MAF for CBs and PBs are shown in Figure 2. As expected, the imputation accuracy was lower for SNPs with lower MAF, and increased with MAF. The most pronounced increase in imputation accuracy was for MAF from the 0.01 to 0.10 for CBs and from 0.01 to 0.05 for PBs (Figure 2). For MAF larger than 0.10 for CBs and 0.05 for PBs, Beagle allelic DR^2^ reached a plateau at about 0.15 for both CBs and PBs. When we performed filtration on Beagle allelic DR^2^, most SNPs with a very low MAF (<0.01) were removed. Also, the average imputation accuracy was higher for PBs compared with CBs, which is most likely due to the higher genetic relationships between the sequenced pigs (60 Duroc males) and the PBs (Duroc females) compared with CBs. Moreover, CBs receive alleles from two other purebred parental lines and these lines are not represented in the reference panel for imputation.

The average animal-specific imputation reliability across the 55 sequenced PBs (only 55 individuals were both genotyped and sequenced) for SSC18 was 0.94 using Beagle V5.0 and 0.91 using FImpute (Supplementary Figure S3). Since the imputation accuracies produced by Beagle V5.0 were larger than FImpute for all analyses, be it only slightly, we used the imputed data from Beagle V5.0 in all subsequent analyses.

### Two-step imputation accuracy

**FIGURE 3:**
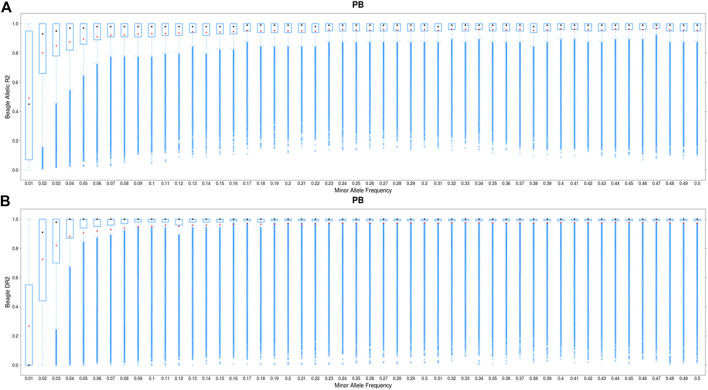
Boxplot showing the imputation accuracy (allelic DR^2^) to whole-genome sequence (WGS) *versus* minor allele frequency (MAF) for PBs using a one-step imputation procedure from 50K to WGS **(A)** and a two-step imputation procedure, from 50K to 660K to WGS **(B)**. The *x*-axis represents different classes of MAF (ranging from 0.01 to 0.5, with the steps of 0.01), and *y*-axis shows the imputation accuracies. The red and black dots are the mean and median of imputation accuracies across individuals in each MAF class. Average DR^2^ across the genome is 0.966 and 0.985 for the one-step and two-step imputation procedure, respectively.

For all chromosomes, the mean imputation accuracy (Beagle allelic DR^2^) was higher (0.90) for one-step imputation approach compared with the two-step imputation approach (0.85) (Supplementary Figure S4). After filtering Beagle allelic DR^2^ < 0.8, the mean imputation for the two-step approach was slightly larger than those obtained from the one-step procedure (Supplementary Figure S4). Figure 3 compares the imputation accuracy (Beagle allelic DR^2^ > 0.8) in one-step (50K to WGS) and two-step imputation (50K to 660K to WGS) procedures, which are plotted against MAF. As shown, the imputation accuracy of low MAF SNPs (MAF <0.02) remains challenging. The average allelic DR^2^ across the genome was 0.996 for one-step approach and 0.985 for two-step approach. Due to very small difference in imputation accuracies between the two approaches, we performed the GWAS analyses only for the imputed variants from the one-step method.

### Variance component estimates

Variance components and heritability estimates obtained from different datasets (CBs, PBs, and Combined dataset) for each color trait are in Table 6. Generally, the heritability estimates were low to high across the 18 meat color traits, and ranged from 0.20 ± 0.06 (GMCOLB) to 0.57 ± 0.09 (TMCOLA) for CBs, from 0.07 ± 0.06 (QFCOLB) to 0.43 (0.10) (GMCOLA) for PBs. When the Combined dataset was used, since the heterogeneous genetic and residual variances were fitted in the model, the heritability for CBs and PBs were estimated by the model separately and the heritabilities ranged from 0.17 ± 0.06 for GMCOLB to 0.60 ± 0.09 for FMCOLL in CBs and from 0.08 ± 0.05 for QFCOLL to 0.37 (0.10) GMCOLA in PBs.

### Genome-wide association studies for pork color traits

Putative family stratifications were accounted for the GWAS analyses by incorporating the full genomic covariance among animals. Lambda ranged from 0.77 for ICOLB in Combined dataset (61K CBs plus 50K PBs) to 1.00 for FMCOLB in sequenced PBs, and the mean lambda across all traits was 0.91 (results not shown), suggesting that any potential bias and any major effect of population stratification was taken into account in the GWAS analyses. For all traits for which the QTL regions were found and for all datasets (CBs, PBs, and Combined dataset), lambda increased slightly as SNP density increased (Table 7). This shows that the inflation of *p*-values is lower when WGS data was used compared with the medium-density SNP panel.

**TABLE 7:** Descriptive statistics of results of the GWAS for the pork colors with detected associated regions in at least one of the datasets at FDR >0.1 (CBs, PBs, Combined dataset) using different SNP densities and imputed whole-genome sequence (WGS).

CB								
	61K				WGS			
Trait	Number of significant SNPs	Number of QTL regions	Threshold	Genomic inflation factor	Number of significant SNPs	Number of QTL regions	Threshold	Genomic inflation factor
TMCOLA	2	1	5.29	0.88	396	1	5.27	0.86
GMCOLB	1	1	6.09	0.90	183	1	5.68	0.91
Total number of QTL/significant SNPs	3	2	-	-	579	2	-	-

PB								
	50K				WGS			
Trait	Number of significant SNPs	Number of QTL regions	Threshold	Genomic inflation factor	Number of significant SNPs	Number of QTL regions	Threshold	Genomic inflation factor
FMCOLB	6	1	4.99	0.98	1,363	5	4.86	1.00
FMCOLL	16	6	4.36	0.91	2,034	12	4.70	0.94
ICOLB	19	2	4.27	0.96	933	2	** **4.98	0.98
Total number of QTL/significant SNPs	41	9	-	-	4,330	19	-	-

Combined Dataset								
	61K + 50K				WGS			
Trait	Number of significant SNPs	Number of QTL regions	Threshold	Genomic inflation factor	Number of significant SNPs	Number of QTL regions	Threshold	Genomic inflation factor
FMCOLA	6	4	4.98	0.90	2,773	9	4.57	0.95
FMCOLB	5	2	4.90	0.93	2,645	12	4.60	0.96
GMCOLB	8	3	4.61	0.87	2,593	9	4.60	0.97
ICOLB	8	2	4.57	0.77	3,341	7	4.49	0.85
Total number of QTL/significant SNPs	27	11	-	-	11,352	37	-	-
Total number of QTL/significant SNPs for all datasets (CBs, PBs, Combined dataset)	71	22	-	-	16,261	58	-	-

CB, crossbred; PB, purebred; SNP, single nucleotide polymorphism; WGS, whole-genome sequence; QTL, quantitative trait loci.

**FIGURE 4:**
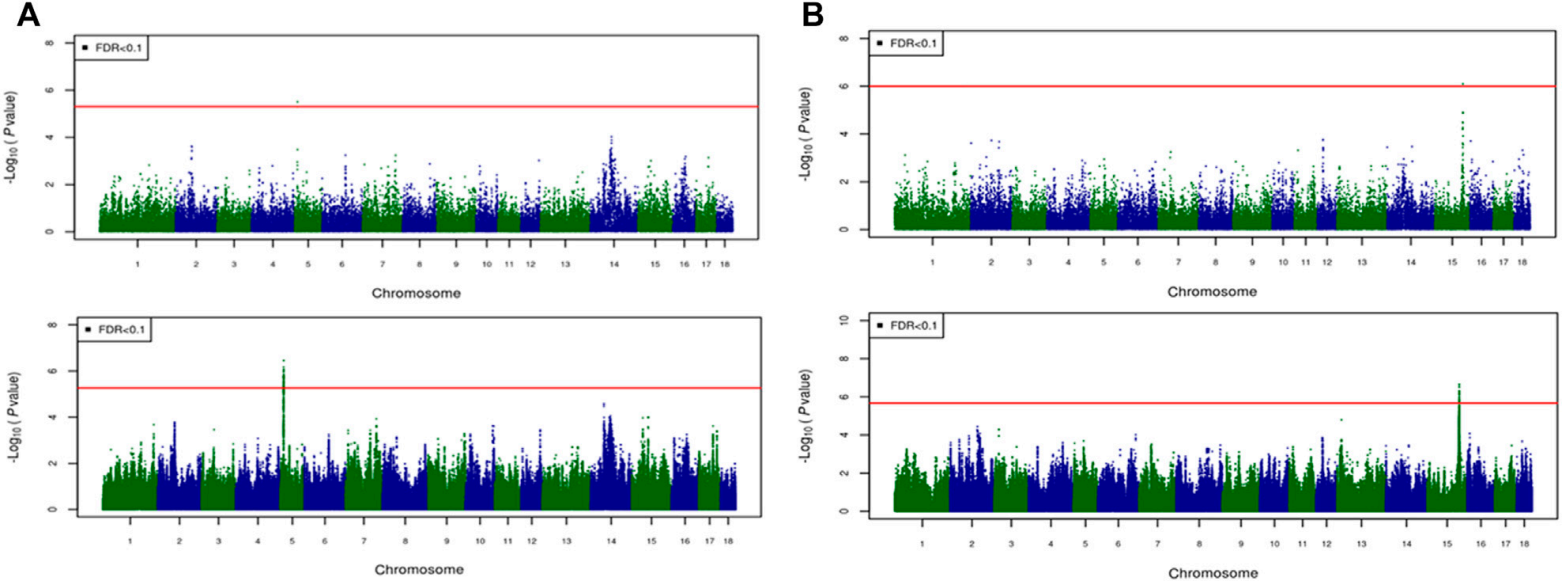
Associated regions detected by GWAS for crossbred pigs. Manhattan plots for: **(A)** TMCOLA and **(B)** GMCOLB using a 61K medium-density panel (top Figure) and WGS (bottom Figure). The -log10 *p*-values of single-SNP association along the entire genome are plotted against the genomic position of SNPs along the 18 autosome chromosomes. The SNPs associated with the corresponding traits exceeded the significance threshold at false discovery rate (FDR) of 0.1, having significant effects.

**FIGURE 5:**
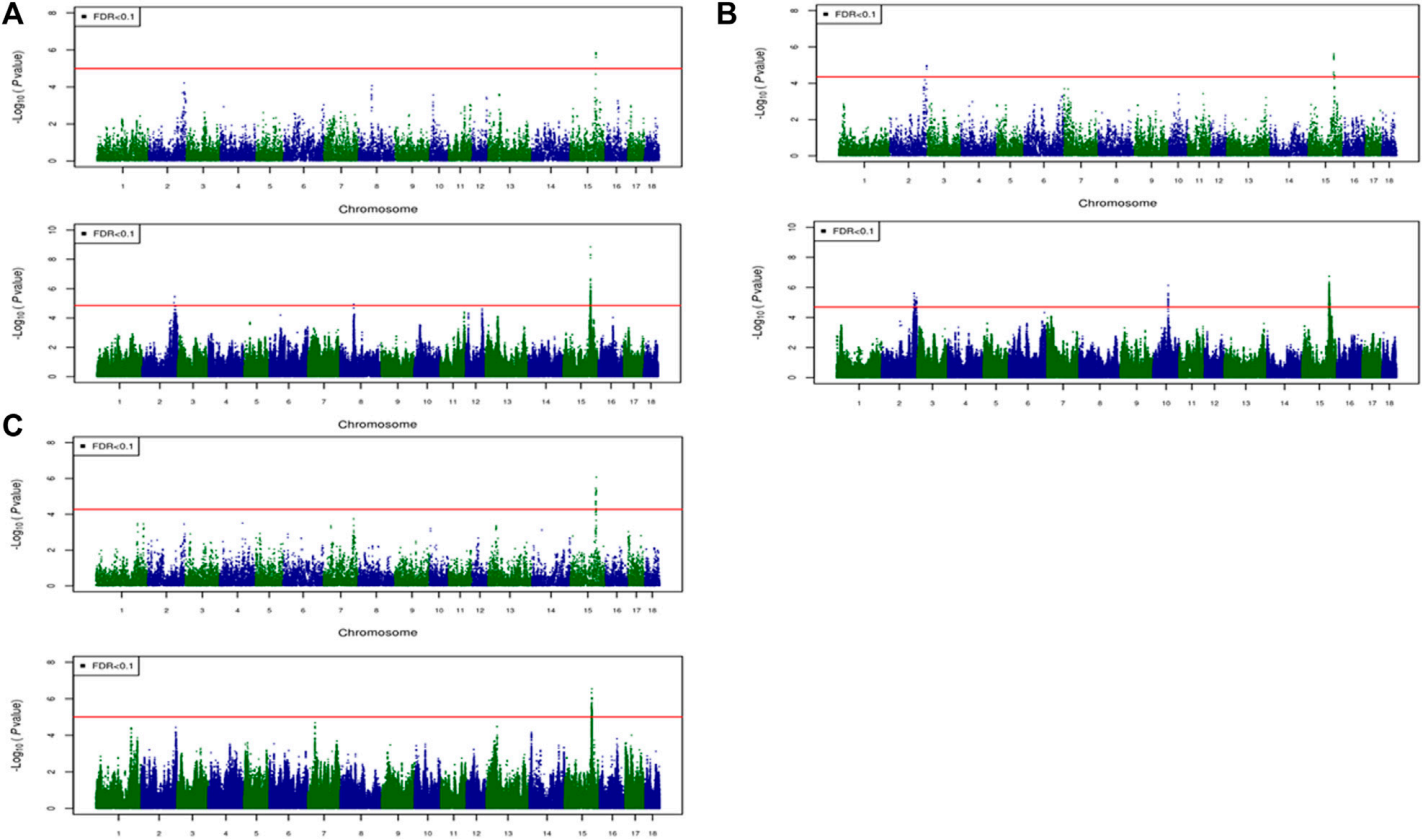
Associated regions detected by GWAS for purebred pigs. Manhattan plots for: **(A)** FMCOLB, **(B)** FMCOLL, and **(C)** ICOLB using a 50K medium-density panel (top Figure) and WGS (bottom Figure). The -log10 *p*-values of single-SNP association along the entire genome are plotted against the genomic position of SNPs along the 18 autosome chromosomes. The SNPs associated with the corresponding traits exceeded the significance threshold at false discovery rate (FDR) of 0.1, having significant effects.

**FIGURE 6:**
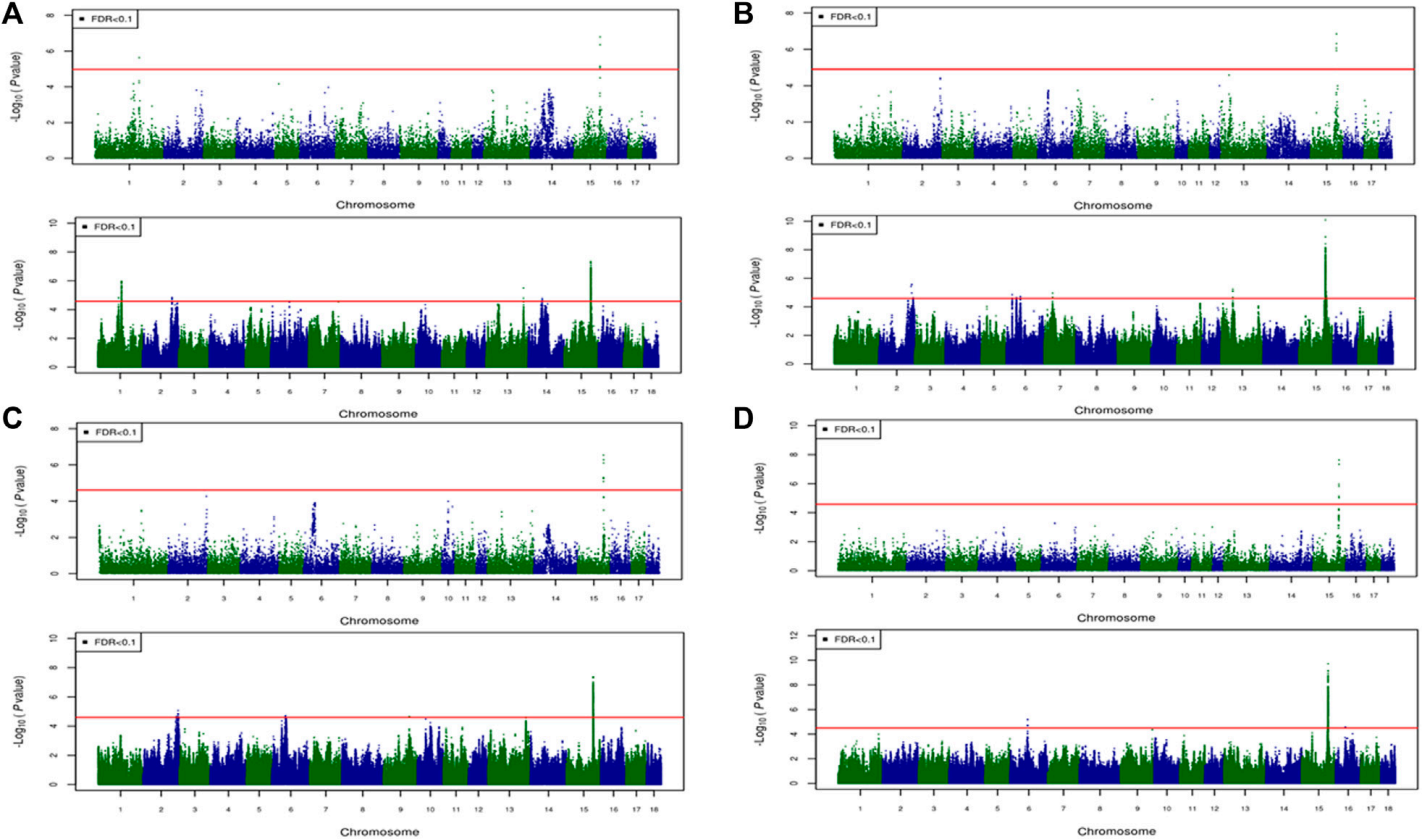
Associated regions detected by GWAS for combined crossbred and purebred pigs. Manhattan plots for: **(A)** FMCOLA **(B)** FMCOLB **(C)** GMCOLB, and **(D)** ICOLB using a combined (61K + 50K) medium-density panel (top Figure) and WGS (bottom Figure). The -log10 *p*-values of single-SNP association along the entire genome are plotted against the genomic position of SNPs along the 18 autosome chromosomes. The SNPs associated with the corresponding traits exceeded the significance threshold at false discovery rate (FDR) of 0.1, having significant effects.

For most color traits (16 color traits for CBs, 12 color traits for PBs, and 11 color traits for Combined dataset), zero associated regions were detected at FDR of 0.1. Total number of significant SNPs, number of QTL regions, FDR threshold, and genomic inflation factor values are given in Table 7. Generally, more QTL were detected for traits of PBs than those of CBs at FDR = 0.1. However, when we used a more relaxed FDR threshold >0.1 and up to 0.4, suggestive significant SNPs were detected for some traits in different datasets, i.e., for FCOLA (PBs), FCOLL (PBs), QFCOLA (CBs, PBs, and Combined dataset), FMOCLA (CBs and PBs), FMOCLB (CBs), FMCOLL (Combined dataset), GMCOLA (PBs and Combined dataset), GMCOLB (PBs), GMCOLL (CBs), and ICOLB (PBs) (results not shown). For CBs, significant SNPs were identified for TMCOLA and GMCOLB (Table 7; Figure 4), and for PBs, the associated SNPs were found only for FMCOLB, FMCOLL, and ICOLB (Table 7; Figure 5). For the Combined dataset, we found the associated variants for more traits including FMCOLA, FMOCLB, GMCOLB, and ICOLB (Table 7; Figure 6).

For all traits and using the medium-density panels, 22 QTL regions containing 71 significant SNPs at a genome-wide FDR of 0.1 were detected, whereas 58 QTL regions comprising 16,261 significant SNPs were detected at the same significance level using WGS data (Table 7; Figures 4–6). The twenty two regions detected by medium-density panels overlaped with those detected by WGS. The number of QTL regions (2 using 61K and 2 using WGS) and significant SNPs (3 using 61K and 579 using WGS) were lowest for CBs using both SNP panel densities (61K and WGS), while the number of QTL regions (11 using 50K and 37 using WGS) and significant SNPs (11,352 using WGS) were highest when the Combined dataset was used for GWAS for both panel densities, except for the number of significant SNPs detected by PBs using 50K data which was highest (41 SNPs) compared with CBs (3 SNPs) and combined data (27 SNPs) (Table 7).

Generally, the number of QTL regions increased with increasing panel density mainly for PBs and the Combined dataset, and did not change for CBs. For instance, for GMCOLB, the number of detected QTL region was only 1, regardless of what SNP density (61K or WGS) were used. For PBs, the additional QTL regions were located on SSC2 at 142.79–144.77 Mb and on SSC8 at 34 Mb, for FMCOLB, and on SSC10 at 38.13–38.29 Mb for FMCOLL (Table 8). Of all the new detected QTLs by WGS in PBs, the strongest new significant QTL was identified on SSC10 for FMCOLL (Figure 5). For the Combined dataset, the novel QTL regions identified by WGS compared with the medium-density SNP panel are given in Table 8 (also see Figure 6). Of all the new detected QTLs by WGS, the strongest new significant QTLs were identified on SSC1 for FMCOLA. Moreover, the total number of associated SNPs increased by increasing SNP density from 61K or 50K to WGS (Table 7; Figures 4–6). For example, it increased from 3 to 579 for CBs, from 41 to 4,330 for PBs, and from 27 to 11,352 for the Combined dataset.

**TABLE 8:** Novel genomic regions detected by WGS in PB pigs and in Combined dataset (combined CBs and PBs).

Trait	Number of QTL regions and their genomic region in mega base pairs (Mb)
PB	
FMCOLB	2 QTL regions on SSC2 (142.79 and 144.77–144.77). 1 QTL region on SSC8 (34 Mb)
FMCOLL	1 QTL region on SSC10 (38.12–38.28)
Combined dataset	
FMCOLA	1 QTL region on SSC2 (134.56–134.74). 1 QTL region on SSC13 (194.82–194.82). 1 QTL region on SSC14 (44.85–45.02)
FMCOLB	3 QTL regions on SSC2 (142.79, 144.77–144.81, and 148.00). 3 QTL regions on SSC6 (19.22–19.26, 57.29–57.30, and 32.18–32.22). 1 QTL region on SSC7 (23.73–23.85). 1 QTL region on SSC13 (24.73–24.74)
GMCOLB	2 QTL regions on SSC2 (144.89–144.95 and 149.09–149.12). 2 QTL regions on SSC6 (57.25 and 52.57–52.62). 1 QTL region on SSC9 (119.68–119.69). 1 QTL region on SSC13 (195.48)
ICOLB	1 QTL region on SSC6 (73.97–74.05). 1 QTL region on SSC16 (22.84)

PB, purebred; SNP, QTL, quantitative trait loci; Mb, megabyte; SSC, *sus scrofa.*

For all datasets (CBs, PBs, and Combined dataset) and for all SNP panel densities (61K, 50K, and WGS), the majority of the significant SNPs were on SSC15 (Figures 4–6). For WGS, most of the significant SNPs were on SSC15 (93.17%), following by SSC5 (2.44%), and SSC2 (2.43%). The genomic location of the peak on SSC15 (across the traits) was between 119.57 and 122.50 Mb and between 119.56 and 123.56 for medium-density SNP panel and WGS, respectively. The position of the majority of SNPs within this window was the same between the medium-density and WGS data. For medium-density SNP panels, of the 71 significant SNPs, almost all of the significant SNPs were on SSC15 (∼88%), except for 9 significant SNPs. Those 9 SNPs were: five SNPs detected by PBs for FMCOLL on SSC2 at 147.22–150.43 Mb, 1 SNP detected by the combined data for FMCOLA on SSC1 at 164.72 Mb, 1 SNP detected by the combined data for GMCOLB on SSC2 at 144.95 Mb, and finally 2 SNPs detected by PBs for TMCOLA on SSC5 at 9.41–9.44 Mb. Based on these results, using the medium-density SNP panels, only a few new QTL regions and SNPs were detected by Combined dataset compared with BPs and CBs. However, using WGS data, many more new QTL regions and SNPs were detected by only Combined dataset, and not detected by PBs and CBs, suggesting that increasing both the sample size and SNP density together improves identification of associated genomic regions.

**FIGURE 7:**
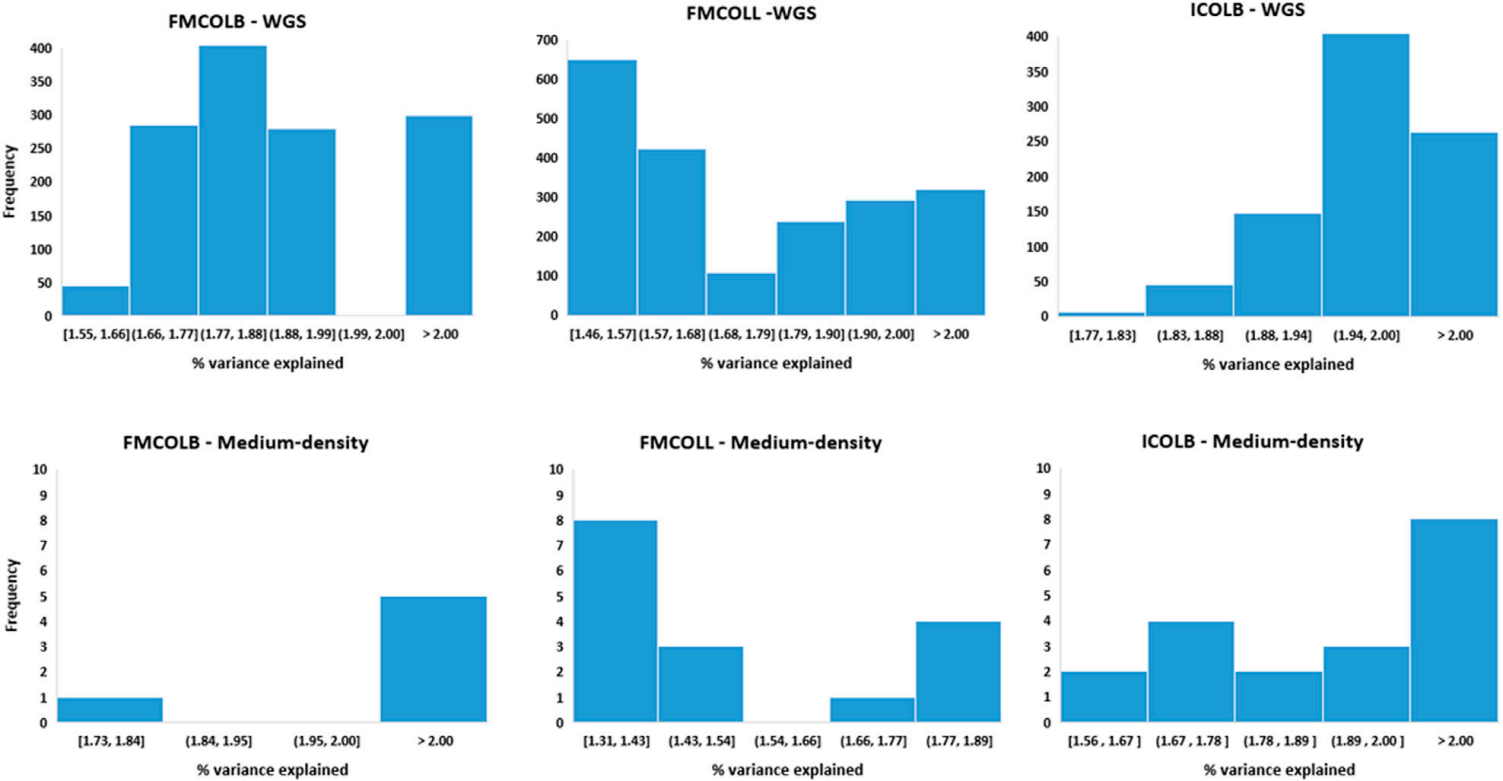
Distribution of the percentage of phenotypic variance explained by the most significant SNPs identified using different SNP densities (WGS (top row) and medium-density panels (bottom row)) for pork color traits (FMCOLB, FMCOLL, and ICOLB) in PBs.

Besides the increase of the number of QTL regions with WGS, the percentage of the phenotypic variance explained by the most significant SNPs also increased by WGS compared with medium-density panels (Figure 7). Figure 7 shows the distribution of the percentage of phenotypic variance explained by the most significant SNPs identified using both WGS and medium-density panels (50K) for three pork color traits (FMCOLB, FMCOLL, and ICOLB) in PBs. For these three traits, the number of SNPs that explained more than two percent of phenotypic variance increased from 5 to 299 for FMCOLB and from 0 to 321 for FMCOLL, and from 8 to 263 for ICOLB, when WGS was used for GWAS compared with using 50K for GWAS. The threshold 2% was chosen, because the maximum percentage of variance explained by the significant SNPs that exceed the FDR of 0.1 were ∼3% and we therefore chose an arbitrary threshold lower than 3%.

### Candidate genes identified by functional analysis

Candidate genes (and their functions) located on significant regions and/or nearby regions identified by WGS for meat color traits in different populations (crossbreds (CBs), purebreds (PBs), combined CBs and PBs) are given in Table 9. Since most of the significant SNPs for most traits are located on SSC15, for simplicity only the results of the functional analyses on SNPs detected on SSC15 are explained. For all traits, the significant SNPs span a region from 119.56 to 123.56 Mb on SSC15. The genes on this region are in Table 9. Many of these genes such as *PRKAG3* have been previously reported by Zhang et al. (2015) to be associated with pork pH and color. Some of the genes located on this region including *CNOT9, PRKAG3, CDK5R2, VIL1, TTLL4, CTDSP1, SLC11A1, ZFAND2B, USP37, RNF25, STK36, FEV, WNT6, IHH, WNT10A, NHEJ1, TMBIM1* are involved in the regulation of protein phosphorylation processes, proteolysis, intracellular transduction, and negative regulation of cell communication. In addition, *CATIP* and *ARPC2*, *VIL1* and *BCS1L* are involved in actin filament organization. There is evidence in the literature that meat color stability is inversely related to the phosphorylation of sarcoplasmic proteins (Mato et al., 2019; Li et al., 2020). An example of visualized gene network for the genes on SSC15 of ICOLB (region: 119.5–122.5 Mb) which shows the involved biological process is given in Supplementary Figure S5.

**TABLE 9:** Candidate genes located on significant regions and/or nearby regions identified by whole-genome sequence (WGS) for meat color traits in different populations (crossbreds (CBs), purebreds (PBs), combined CBs and PBs).

	Trait	Chromosome^a^	Physical position (Mb)^b^	Candidate genes (gene functions)
CB	TMCOLA	SSC5	115.3–117.4	*FBX O 7*, *TIMP3*, *PWP1*, *RAC2*, *EIF3D*, *KCTD17* (Negative regulation of protein phosphorylation), cellular metal ion homeostasis (*KCTD17, PVALB*), Apolipoprotein L3-like, *RBFOX2, FBX O 7*
	GMCOLB	SSC15	120.6–120.9	*CATIP, ARPC2, VIL1* (Actin filament organization), *CNOT9*, *PLCD4, PRKAG3*, *DNAJB2, ZFAND2B, CNPPD1, INHA, CDK5R2, STK16, TTLL4, USP37, CTDSP1*, *SLC11A1*
PB	FMCOLB	SSC2	142.7–144.7	*ARHGAP26* (MAPK cascade and protein transport)
		SSC15	120.1–120.9	*ARPC2*, *CATIP*, *BCS1L*, *VIL1* (Actin filament polymerization)*, PRKAG3, TTLL4, CTDSP1, USP37, SLC11A1* (Protein modification processes and protein phosphorylation)
	FMCOLL	SSC15	120.1–122.5	The same genes as previously described for FMCOLB.
	ICOLB	SSC15	119.5–122.5	*CNOT9,PRKAG3,CDK5R2,VIL1,TTLL4, CTDSP1, SLC11A1, ZFAND2B, USP37, RNF25, STK36, FEV, WNT6, IHH, WNT10A,NHEJ1, MBIM1* (Regulation of protein phosphorylation processes, proteolysis, intracellular transduction, and negative regulation of cell communication)*, CATIP* and *ARPC2*, *VIL1*, *BCS1L* (actin filament organization)
Combined dataset	FMCOLA	SSC1	163.9–166.8	*MEGF11, U2, DIS3L, TIPIN, SCARNA14, MAP2K1, SNAPC5, RPL4, SNORD18, ZWILCH, LCTL, SMAD3, SMAD6, AAGAB, IQCH, C15orf61, MAP2K5, SKOR1, U6, PIAS1, CALML4, CLN6, FEM1B, ITGA11,* and *COR O 2B* (positive regulation of proteolysis, negative regulation of cell cycle, and regulation of transforming growth factor beta receptor signaling pathway)
		SSC15	120.1–120.9	The same genes as previously described for FMCOLB (purebreds)
	FMCOLB	SSC15	120-123.5	The same genes as previously described for FMCOLB (purebreds)
	GMCOLB	SSC2	144.7–150.9	nuclear receptor subfamily 3 group C member 1 (*NR3C1*), phosphodiesterase 6A (*PDE6A*), serine peptidase inhibitor, Kazal type 6 (*SPINK6*), *ARHGAP26*
		SSC6	52.57–59.13	Zinc finger protein 836-like, zinc finger protein 347 gene, NLR family pyrin domain containing 7, *PRK2*, *STRN4*
		SSC15	119.98–120.92	*PNKD, CNOT9, PLCD4, TMBIM1*, zinc finger protein 142 and *SLC11A1, TNS1, RUFY4, ARPC2, GPBAR1, AAMP, CATIP, CTDSP1, VIL1, USP37, BCS1L, RNF25, STK36, TTLL4, CYP27A1, PRKAG3, WNT6* and *WNT10A*
	ICOLB	SSC6	73.93–74.04	kazrin, periplakin interacting protein (*KAZN*) gene
		SSC15	119.55–120.92	The same genes as previously described for GMCOLB (Combined dataset)
		SSC16	22.59	*WDR70*

b This is the physical position in Mb and their nearby regions where the candidate regions were found (See Materials and Methods).

a If a significant region was not reported, no genes were found in that region. CB, crossbred; PB, purebred; Mb, megabyte; SSC, *sus scrofa.*

## Discussion

In this study, we first assessed the imputation accuracy to WGS for two pig populations; CBs and PBs, using a small reference population of 60 sequenced PB key ancestors (Duroc males). Then, using the imputed WGS, we investigated whether the use of WGS data in a GWAS for pork color traits will improve the identification of the associated regions with respect to the extended QTL regions and/or detection of novel QTL regions in a sequenced-based GWAS relative to a medium-density SNP panel. The superiority of WGS over SNP panels is because of the existence of causal variants (rare variants responsible for phenotype variation) and rare variants with low LD with the SNPs on a medium-density panel (which most have moderate MAF), as the variance explained by these causal and rare variants can be better captured by WGS. Moreover, due to the relatively small size of our CB and PB populations (lower than 1,000 individuals per population), the Combined dataset was used in a GWAS, to assess whether enlarging the sample size will improve the potential advantage of WGS and enhance the power of detecting QTLs. Our imputation results showed a relatively high imputation accuracy obtained by Beagle V5.0 for both PBs (0.97) and CBs (0.91) after filtering the less accurate imputed genotypes (<0.8). Of the 18 pork colors, using different datasets, the genetic associations were identified only for a few traits (Table 7; Figures 4–6), and we did not detect any associated regions for most traits, regardless of panel density and dataset. WGS detected additional novel genomic regions for a few traits and with larger sample size (Combined dataset) (Table 8), the added value of WGS was more for detecting novel regions compared with SNP panel arrays. In the following sections, first, the factors influencing imputation accuracies are discussed, and then, the impact of using WGS data on GWAS results are discussed in detail.

### Factors influencing imputation accuracy

Several factors influence the accuracy of imputation. These include the size of the reference population, the level of genetic relationship between the reference and validation population (Hickey et al., 2011; Heidaritabar et al., 2015), MAF of the SNPs to be imputed (Heidaritabar et al., 2015; Bouwman et al., 2018), the program used for imputation (Bouwman et al., 2018; Bolormaa et al., 2019), and the density of validation population (Heidaritabar et al., 2015). The biggest challenge when imputing to WGS data is the imputation of the rare variants with low frequency. Figure S2 shows that of approximately 12 million called SNPs on SSC1 to SSC18, about 28% have a frequency less than 0.05 (Supplementary Figure S2). Due to the existence of this large proportion of rare SNPs, it is crucial to impute these variants as accurately as possible. To achieve the highest possible imputation accuracy for rare SNPs, several things can be done including careful selection of the reference individuals, appropriate imputation programs (Calus et al., 2014; Bouwman et al., 2018), and sequencing a sufficient number of animals, (Calus et al., 2014). The 60 Duroc males we chose for sequencing were key ancestors and jointly captured the maximum proportion of genetic variation present among the PBs. This is most likely reason that we achieved relatively high average imputation accuracies (average across all chromosomes and across all MAF) for both CBs (0.80) and PBs (0.90) (Table 3). Moreover, for low MAF SNPs (≤0.05), the average imputation accuracy ranged from 0.35 (when MAF was 0.01) to 0.65 (when MAF was 0.05) in CBs, and ranged from 0.5 to 0.9 in PBs, when MAF was 0.01 and 0.05 respectively (Figure 2). Even though the panel density of PBs is lower than CBs (50K *versus* 61K), PBs imputation accuracies are higher, which is likely due to the larger genetic relationships between the 60 reference sequenced pigs and the female PBs in the validation, as both population are Duroc and results in sharing more and longer haplotypes between the two populations (Hickey et al., 2011), while the CB population include the three-way cross between Duroc boars and Landrace-Yorkshire sows, and therefore, there is lower genetic relationship between the 60 Duroc boars and the CB population. Several studies have investigated the imputation of low MAF SNPs when imputing to the WGS in different species such as dairy cattle (van Binsbergen., 2017), beef cattle (Froberg Brøndum et al., 2014), pigs (Yan et al., 2017; Bouwman et al., 2018; Ros-Freixedes et al., 2019), sheep (Bolormaa et al., 2019), and found a poor imputation accuracy for low MAF SNPs. For example, Ros-Freixedes et al. (2019) reported imputation accuracy of 0.79 for MAF between 0.005 and 0.028 (*n* = 2,111), and 0.93 for MAF above 0.028 (*n* = 25,968) with simulated data, and for accuracy ranging from 0.51 (*n* = 11,312) for MAF <0.001 to 0.93 (*n* = 89,701) for MAF ≥0.028 in pigs. Even though Ros-Freixedes et al. (2019) used a much larger reference population compared to the 60 individuals in our study, our imputation accuracy from PBs for low MAF SNPs are similar to the values reported by them. Also, Bouwman et al. (2018) used three different imputation programs, and found imputation accuracy ranging from 0.5 to ∼0.83 for SNPs with MAF lower than 0.05, when 168 sequenced pigs were used for imputation. Our imputation accuracy for low MAF SNPs from CBs are within the range reported by Bouwman et al. (2018) (0.35–0.65). Of note is that our measure of imputation accuracy is allelic DR^2^, which is reliability, whereas the measure reported by Bouwman et al. (2018) and Ros-Freixedes et al. (2019) is the correlation between the true genotypes and imputed dosages. Meaning that with conversion of the allelic DR^2^ to correlations, our imputation accuracy becomes even higher (*r* = 0.59 to 0.81 for CBs and r = 0.71 to 0.94 for PBs). This suggests that the overall performance of Beagle V5.0 for imputation of low MAF SNPs was good, even with a small reference population size and small genetic relationship between the CBs and PBs. However, to be more certain about the performance of Beagle V5.0 compared with other imputation programs, we compared imputation accuracies from Beagle V5.0 and FImpute in a leave-one-out cross validation approach (Supplementary Figure S3). The average animal-specific imputation accuracy across 55 pigs was slightly higher for Beagle (0.94) than FImpute (0.91).

Increasing the size of the reference population was more beneficial for imputing rare SNPs compared with more common SNPs for both imputation to the WGS in cattle (van Binsbergen et al., 2014), and imputation from low-to medium-density SNP panel (60K) in layer chickens (Heidaritabar et al., 2015). This is because with a larger reference population, the probability that multiple copies of alleles are present for correct haplotype construction increases and this in turn increases the quality of imputation of low-frequency SNPs. For dairy cattle, it was proposed to sequence not more than 500 individuals, as more than this number only slightly improved the accuracy of imputation accuracy. However, it is generally hard to determine exactly how many more sequenced individuals are required as the reference, and which level of genetic relationship to the validation population is required for minimizing the imputation error rate (Meuwissen et al., 2013). Based on our results, it seems that the low number of sequenced animals, when carefully selected, is only a limiting factor for imputation of low MAF SNPs, as we still obtained reasonable imputation reliabilities for high MAF SNPs. In our analyses, we excluded many of those low MAF SNPs (∼20%) with low accuracy of imputation (Supplementary Figure S2), meaning that some of the causative mutations contributing to the genetic variation of a complex trait may have been removed during the filtration of MAF. If enlarging the reference population is not possible due to high costs of sequencing, an alternative to retaining the low MAF SNPs (potential causative mutations) is to use dosage scores instead of genotypes for downstream analyses such as GWAS, or genomic predictions. Van den Berg et al. (2019) compared the GWAS results of using genotypes with those of dosage scores and found an improvement of QTL detection (56.7 and 26.9% additional QTL regions for their two studied lines), because dosage scores coded as any real value between 0 and 2 accounted for uncertainty of imputation, and therefore all SNPs were used in their analysis. They also found that the most significant SNPs in the QTL regions explained more of the phenotypic variance when using dosage scores compared to using genotypes (Van den Berg et al., 2019).

### Genome-wide association studies using purebred pigs, crossbred pigs and Combined dataset

We did a GWAS for 18 pork color traits in CBs, PBs, and combined data using both SNP panel arrays and WGS and investigated whether the WGS can improve the power of GWAS compared to the medium-density SNP panels. Of the 18 pork colors, using different datasets, we did not detect any associated regions for most traits, regardless of panel density (see Results). The QTL regions were identified (with FDR of 0.1) only for a few traits including TMCOLA and GMCOLB (CBs), FMCOLB, FMCOLL, and ICOLB (PBs) and FMCOLA, FMCOLB, GMCOLB, and ICOLB (Combined dataset). Generally, we identified more QTL regions with WGS (*n* = 58) compared with medium-density SNP panels (*n* = 22). Most of the identified QTL regions with all genotype densities were also reported in other GWAS studies that used the same color traits (Zhang et al., 2015; Yang et al., 2017). The most significant QTL region reported by Zhang et al. (2015) was located on SSC15 spanning 133–134 Mb which explained 3.51%–17.06% of genetic variance for five measurements of pH and some color traits (Minolta color A* and B* for fresh ham and color B* measured on thawed loin muscle). This region is very close to previously reported gene *PRKAG3* controlling both meat pH and color in pigs. Our results are consistent with results of Zhang et al. (2015) and Yang et al. (2017), as this region^6^ on SSC15 was identified by both densities and the three datasets. In the present study, for both WGS and medium-density panels and for most traits, most of the significant SNPs were on SSC15 at 119.57 and 122.50 Mb for WGS and at 119.56 and 123.56 for medium-density SNP panel (see Results). The percentage of phenotypic variance explained by the most significant SNP on SSC15 for different pork color traits and different density panels are shown in Table 10. It should be noted that in the present study, the percentage of variance explained is not cumulative, because variants were tested one at a time (See model 2) in *Materials and Methods*). Thus, the estimated SNP effects of surrounding variants were not independent due to LD. For all traits where the genomic region on SSC15 was significant, the variance explained by the most significant SNP was higher for WGS compared with medium density panels. The added value of WGS for improving the power of GWAS (with respect to the number of identified QTL) have been shown in several species including dairy cattle (Daetwyler et al., 2014; van den Berg et al., 2019), beef cattle (Zhang et al., 2015; Wang et al., 2020), pig (Yan et al., 2017), human (The 1000 Genomes Project Consortium. 2010; Höglund et al., 2019), and tomato (Van Binsbergen et al., 2014). A general speculation for more power of GWAS in denser genome coverage with (WGS) is the presence of causative SNPs and SNPs with higher LD within the data, which improves the power for identification of SNPs with small effects.

**TABLE 10:** **P**ercentage of phenotypic variance explained by **the most significant SNP on SSC15 for the pork color traits at different panel densities and different populations.**

Trait	Population	Panel density	Physical position (Mb)	Percentage of phenotypic variance explained
GMCOLB	CB	WGS	120.72	1.82
GMCOLB	CB	61K	120.71	1.67
FMCOLB	PB	WGS	120.42	3.05
FMCOLB	PB	50K	120.80	2.17
FMCOLL	PB	WGS	120.86	2.37
FMCOLL	PB	50K	120.80	1.89
ICOLB	PB	WGS	120.67	2.59
ICOLB	PB	50K	120.86	2.37
FMCOLA	Combined CB and PB	WGS	120.19	1.16
FMCOLA	Combined CB and PB	Medium-density	120.80	1.05
FMCOLB	Combined CB and PB	WGS	120.42	2.06
FMCOLB	Combined CB and PB	Medium-density	120.80	1.29
GMCOLB	Combined CB and PB	WGS	120.66	1.27
GMCOLB	Combined CB and PB	Medium-density	120.21	1.05
ICOLB	Combined CB and PB	WGS	120.67	1.57
ICOLB	Combined CB and PB	Medium-density	120.70	1.15

CB, crossbred; PB, purebred; Mb, megabyte; WGS, whole-genome sequence. Combined CB and PB means combining crossbred and purebred populations, Physical position (Mb) means genomic position in Megabyte,

When the combined dataset was used for GWAS, many more QTL regions (11 for medium-density panels and 37 for WGS) were identified, suggesting that the added value of WGS was more for detecting novel regions compared with medium-density SNP panels in larger samples. This could be because with the larger sample size, the effect of causative mutations on polygenic quantitative traits might be estimated more accurately. Also, for the Combined dataset, we filtered the imputed genotypes based on the allelic DR^2^, meaning that some of the imputed SNPs excluded in CBs analyses (3,016,352 SNPs) due to imputation accuracy less than 0.8 were included in the Combined dataset GWAS analysis, and yet the power of GWAS improved. This shows that the imputation error rate is not really a limiting factor for GWAS. Similar results are shown by Van Binsbergen et al. (2014) where they found that despite their relatively low imputation accuracy (average correlation of 0.34 between true genotypes and allele dosage) in tomato WGS data, the power of a GWAS can still be improved. They reported that more significant SNPs (>65 SNPs in 9 regions) were found in the GWAS using the imputed WGS compared to using the low-density SNP arrays (no significant SNPs). They argued that as long as the squared imputation accuracy (allelic DR^2^ in our study) is higher than the expected LD between the SNPs on the lower density panel (50K and 61K in our study) and the SNPs in the WGS data, imputation is advantageous, as more information is still added by imputation (Van Binsbergen et al., 2014). Average LD between the imputed sequenced SNPs located within 2 Mb windows and shorter (on SSC1) was 0.31 and 0.40 for CBs and PBs, respectively, which is lower than the average squared imputation accuracy, which is 0.91 and 0.97 for the corresponding populations (see Table 3). This may explain why the imputed sequence data improved the QTL detection through a GWAS. Moreover, van den Berg et al. (2019) found that although their imputation from 80K to 660K to WGS in pig populations resulted in poor imputation accuracy (Beagle allelic DR^2^ in their study ranged from 0.39 to 0.49 and from 0.83 to 0.93, before and after variant filtrations), they still found that using imputed WGS instead of a lower density SNP panel increased the number of detected QTL (48.9 and 64.4% more for their different lines) and the estimated proportion of phenotypic variance explained by these QTL (van den Berg et al., 2019). Also, Heidaritabar et al. (2015) found that the average allelic DR^2^ (before quality control) from the 60K SNP panel to WGS imputation in layers was 0.64, but they still observed an increase of prediction accuracy of 1% using WGS compared with 60K for number of eggs. All these results show that most likely the accuracy of the imputed genotypes is not a limiting factor for GWAS and genomic predictions.

### Functional analyses

We detected several candidate genes for the color traits in CBs, PBs and Combined dataset. For most color traits, a region spanning from 119.5 to 123.5 Mb on SSC15 was consistently detected. Some of the genes located on this region including: *ciliogenesis-associated TTC17-interacting protein* (*CATIP*)*, villin-1 (VIL1), protein kinase AMP-activated non-catalytic subunit gamma 3 (PRKAG3), tubulin tyrosine ligase like 4 (TTLL4), ubiquitin specific peptidase 37 (USP37), CTD small phosphatase 1 (CTDSP1)* and *solute carrier family 11 member 1* (*SLC11A1*) were consistently detected for all color traits reported here, hence they were considered the best candidates’ genes in the QTL region for the color traits. Genes such as *VIL1, PRKAG3, TTLL4,* and *SLC11A1, USP37* have been previously reported to be associated with meat quality, pH and color (Ciobanu et al., 2001; Uimari and Sironen, 2014; Zhang et al., 2015; Verardo et al., 2017). Although *CTDSP1* gene has not been previously associated with meat color traits in pigs, it has been found to be associated with meat color Minolta L* traits in Nellore cattle (Marin-Garzon et al., 2021). The genes reported in this study are involved in actin filament organization, regulation of protein phosphorylation processes, proteolysis, and intracellular transduction. There is evidence in the literature that meat color stability is inversely related to the phosphorylation of sarcoplasmic proteins such as myoglobin (Mato et al., 2019; Li et al., 2021). Meat color is determined by myoglobin concentration as well as the relative content of oxymyoglobin, deoxymyoglobin and metmyoglobin (Zhang et al., 2015; Li et al., 2021). Studies have shown that myoglobin phosphorylation may lead to changes in its secondary structure, therefore reducing myoglobin stability and increasing its autoxidation rate, which further accelerated the accumulation of metmyoglobin (Zhang et al., 2015). Further exploration of these genes and protein phosphorylation pathway will improve our understanding on genetic factors affecting meat quality hence leading to strategies to improve color in pork.

## Conclusion

Use of purebred and crossbred populations genotyped by medium-density panels resulted in relatively high imputation accuracy (0.97 for purebreds and 0.91 for crossbreds after variants quality control) to WGS. Additional QTL regions were detected when using the WGS data compared with a medium-density SNP panels. The performance of WGS relative to the medium-density panels is best when the sample size is the largest (combining cross- and purebreds), suggesting that sample size is a limiting factor to capitalize on the added value of WGS in a GWAS.

## Data Availability

The sequence data was generated on commercial Duroc pigs owned by Hendrix Genetics, Hypor. Data may be available from authors upon reasonable request and authorization from the company.

## Footnotes

1 Only our CB animals with 61K single nucleotide polymorphisms (SNP) panel were used by Zhang et al. (2015) and Yang et al. (2017). Both authors used the same pork color traits as in the present study.

2 The number of phenotypes vary per trait, ranging from 941 to 954 (See *Methods*).

3 The number of phenotypes vary per trait, ranging from 873 to 891.

4 When we mentioned a medium-density SNP panel throughout the manuscript, we meant 61K and 50K SNP panels.

5 Through the manuscript, we call the combination of CBs and PBs as Combined dataset.

6 Zhang et al. (2015) and Yang et al. (2014) used the Sscrofa 10.2, while we used Sscrofa 11.1. The region 133–134 Mb on SSC15 on Sscrofa 10.2 is the same region on 120–121 Mb on Sscrofa 11.1.

## Supplementary material

Supplementary Figure S1 Multidimensional scaling result for assessing the structure of the population.

Supplementary Figure S2
**(A)** Minor allele frequency (MAF) distribution in the 61K SNP panel of CBs and 50K SNP panels of PBs, and sequence Duroc purebred males. **(B)** Minor allele frequency (MAF) distribution of sequence data (11,946,148 SNPs) after MAF filtration with cut-off threshold = 0.025.

Supplementary Figure S3 Animal-specific imputation accuracy for the 55 sequenced animals, using leave-one-out cross-validation approach.

Supplementary Figure S4 Comparison of imputation accuracy (Beagle allelic DR2) in one-step (50k to WGS) and two-step imputation approach before any filtration **(A)** and after filtering Beagle allelic DR2 > 0.8 **(B)**.

Supplementary Figure S5 Gene network constructed based on the candidate and/or nearest genes to the significant SNPs on SSC15 for ICOLB in PBs. Functionally grouped network with terms as nodes are linked based on their kappa score level (0.4).
