# Supplementary material for: Imputation to whole-genome sequence and its use in genome-wide association studies for pork colour traits in crossbred and purebred pigs
Source: Front Genet. 2022 Oct 11;13:1022681. doi: 10.3389/fgene.2022.1022681 (PMC9593086; doi:10.3389/fgene.2022.1022681)
Supplement: Supplementary file 1 [file Table1.DOCX]

**Supplementary Table S1.** Sequence coverage of whole-genome for the 60 sequenced animals.

| Animal | Sequence coverage | Animal | Sequence coverage | Animal | Sequence coverage |
| --- | --- | --- | --- | --- | --- |
| 1 | 22.3 | 21 | 22.78 | 41 | 24.54 |
| 2 | 24.32 | 22 | 25.43 | 42 | 22.14 |
| 3 | 22.81 | 23 | 23.04 | 43 | 23.33 |
| 4 | 23.26 | 24 | 23.33 | 44 | 23.66 |
| 5 | 21.87 | 25 | 23.27 | 45 | 26.15 |
| 6 | 28.12 | 26 | 20.71 | 46 | 24.89 |
| 7 | 20.72 | 27 | 21.55 | 47 | 22.28 |
| 8 | 19.9 | 28 | 25.43 | 48 | 23.01 |
| 9 | 21.16 | 29 | 23.05 | 49 | 21.97 |
| 10 | 19.72 | 30 | 17.78 | 50 | 23.49 |
| 11 | 20.07 | 31 | 20.96 | 51 | 20.6 |
| 12 | 20.98 | 32 | 20.21 | 52 | 20.38 |
| 13 | 17.58 | 33 | 22.66 | 53 | 19.91 |
| 14 | 20.33 | 34 | 19.24 | 54 | 21.88 |
| 15 | 19.81 | 35 | 24.6 | 55 | 20.48 |
| 16 | 21.35 | 36 | 16.99 | 56 | 19.11 |
| 17 | 20.29 | 37 | 19.71 | 57 | 20.23 |
| 18 | 20.37 | 38 | 19.87 | 58 | 23.35 |
| 19 | 20.42 | 39 | 23.8 | 59 | 19.23 |
| 20 | 19.64 | 40 | 21.53 | 60 | 23.63 |
| Average |  |  |  |  | 21.75 |
