# Supplementary figures and images for: Imputation to whole-genome sequence and its use in genome-wide association studies for pork colour traits in crossbred and purebred pigs
Source: Front Genet. 2022 Oct 11;13:1022681. doi: 10.3389/fgene.2022.1022681 (PMC9593086; doi:10.3389/fgene.2022.1022681)

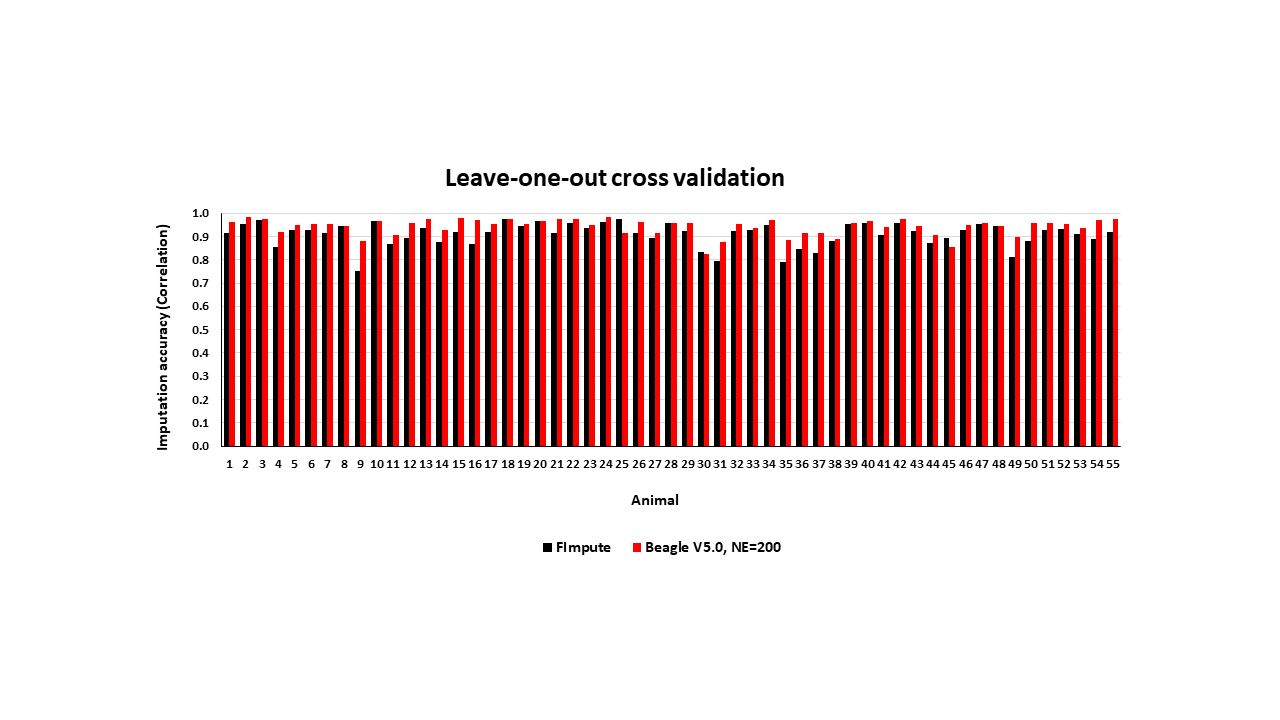

Supplement: Supplementary file 2 [file Image3.TIF]

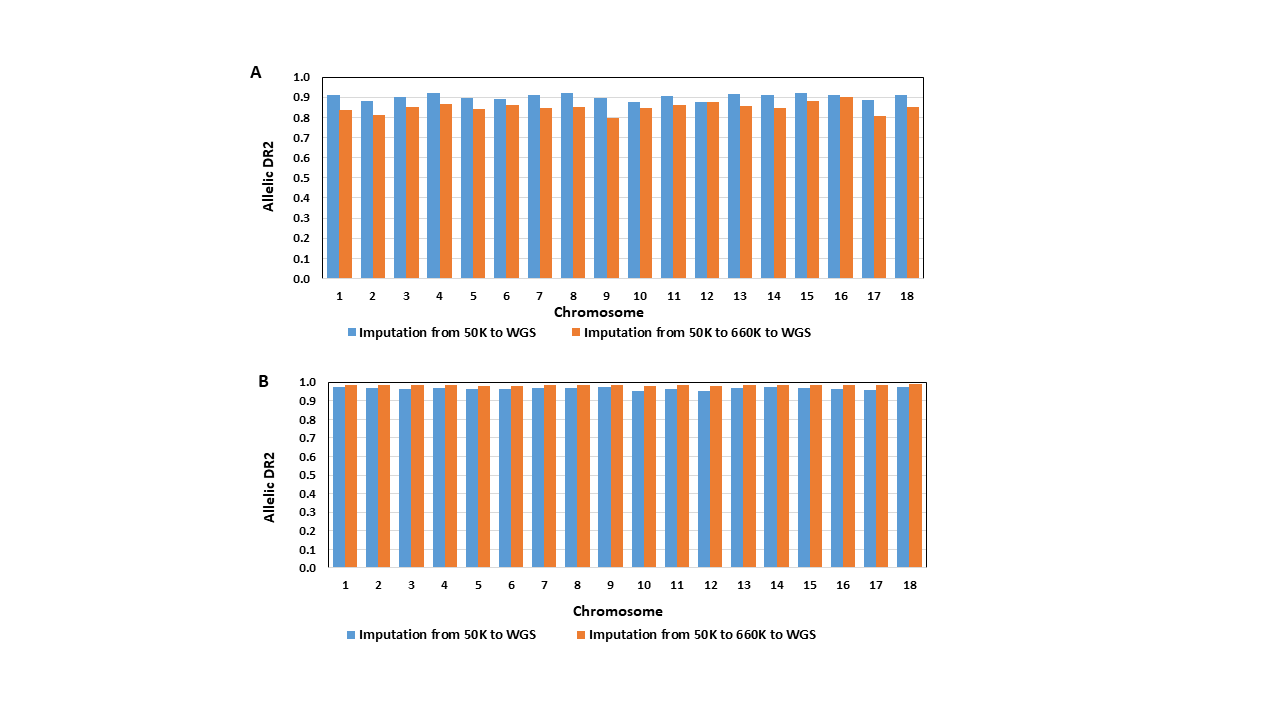

Supplement: Supplementary file 3 [file Image4.TIF]

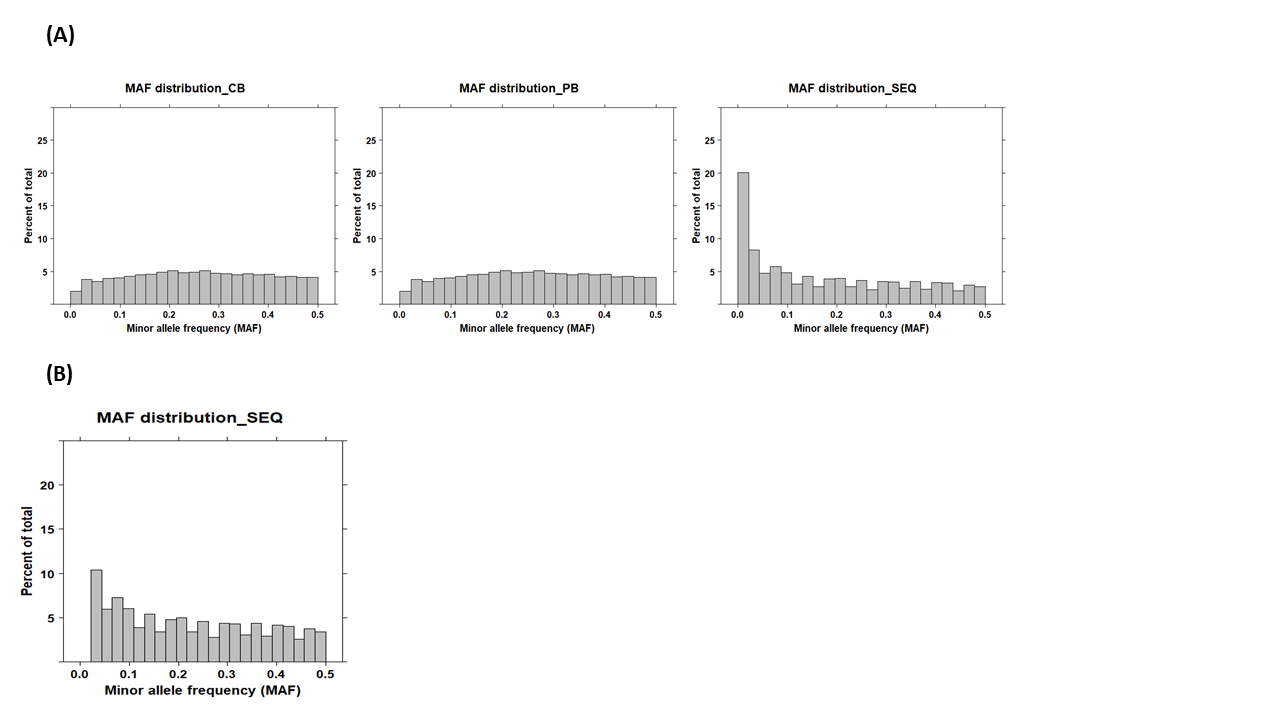

Supplement: Supplementary file 4 [file Image2.TIF]

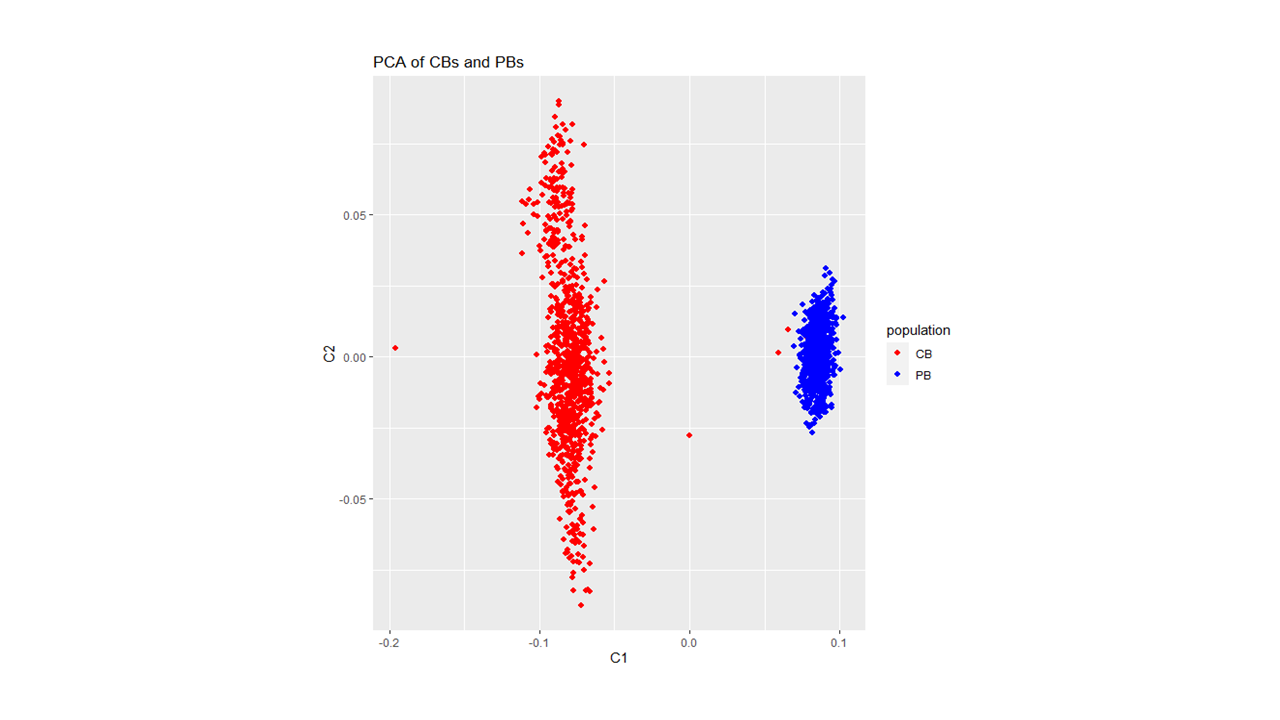

Supplement: Supplementary file 5 [file Image1.TIF]

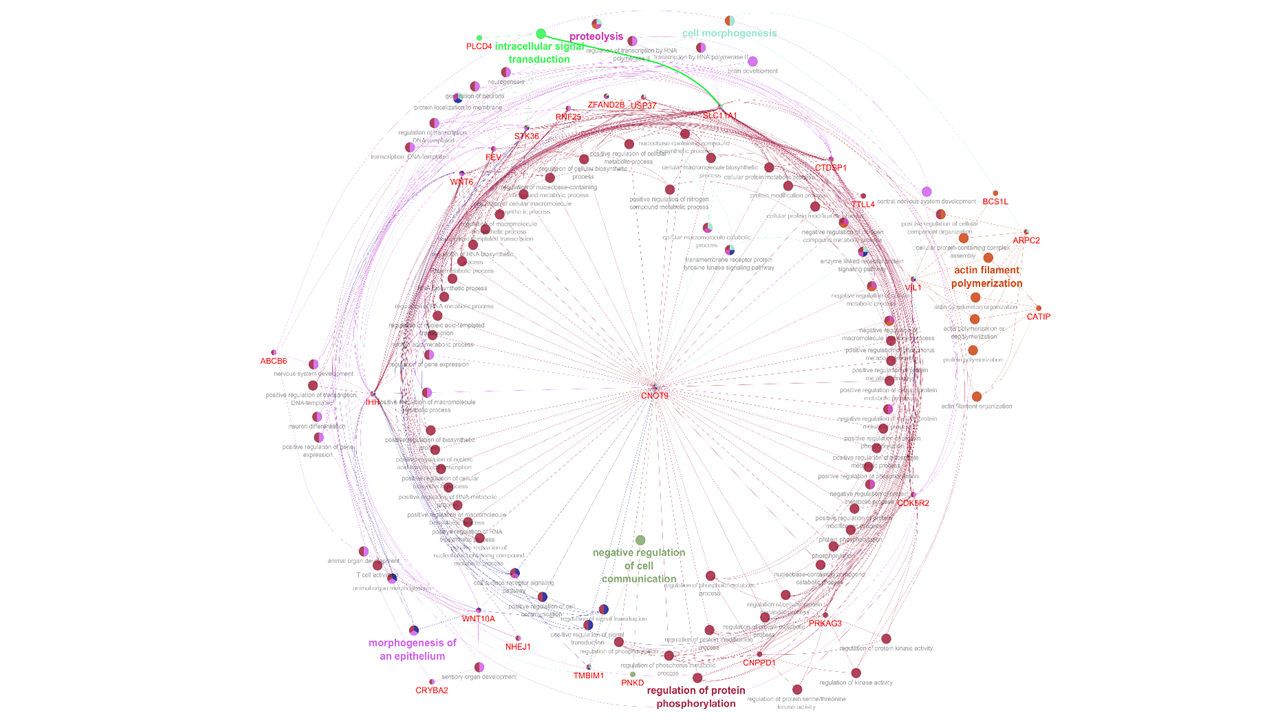

Supplement: Supplementary file 6 [file Image5.TIF]
